# Pathological Copper Overload Reprograms SOD1 Activation via COMMD1 to Promote Senescence and Fibrosis

**DOI:** 10.1002/advs.76391

**Published:** 2026-07-02

**Authors:** Yuqing Liu, Jing Liu, Wenqian Zhou, Yangyang Niu, Yan Zheng, Yiguo Liu, Yingying Zhang, Chen Yu

**Affiliations:** ^1^ Department of Nephrology Tongji Hospital School of Medicine, Tongji University Shanghai China

**Keywords:** cellular senescence, copper homeostasis, copper metabolism MURR1 domain containing 1 (COMMD1), renal fibrosis, superoxide dismutase 1 (SOD1)

## Abstract

Superoxide dismutase 1 (SOD1), a copper‐dependent antioxidant, is essential for redox homeostasis, and its decline drives renal senescence and fibrosis. However, the mechanisms linking profibrotic signaling to SOD1 inhibition remain unclear. Here, we identified a pathological copper‐COMMD1‐SOD1 axis in which intracellular copper overload paradoxically suppressed SOD1 activity. In kidney tissues from chronic kidney disease (CKD) patients and complementary in vivo and in vitro fibrotic models, we consistently observed a reduction in SOD1 activity accompanied by elevated intracellular copper levels. Lowering intracellular copper levels restored SOD1 activity, suppressed reactive oxygen species (ROS) accumulation, and alleviated cell senescence and fibrosis. Mechanistically, pathological copper overload impaired SOD1 homodimerization, the essential final step in its activation. We identified copper metabolism MURR1 domain containing 1 (COMMD1) as a key copper‐sensitive mediator of this process. Copper overload acted upstream, simultaneously upregulating COMMD1 expression and enhancing its binding affinity to SOD1. This enhanced COMMD1‐SOD1 interaction directly disrupted SOD1 homodimer assembly and enzymatic function. Collectively, these findings redefined the regulatory role of copper in SOD1 activity and uncovered a previously unrecognized mechanism by which pathological copper overload paradoxically suppressed SOD1 activity via COMMD1‐dependent disruption of SOD1 homodimerization, providing new insight into the pathophysiology of copper dyshomeostasis‐associated diseases.

## Graphical abstract

This study uncovers a previously unrecognized copper‐COMMD1‐SOD1 regulatory axis, revealing that pathological copper overload paradoxically suppresses SOD1 activity by promoting COMMD1‐dependent disruption of SOD1 homodimerization. These findings redefine the regulatory role of copper in SOD1 biology and provide novel mechanistic insight into the pathophysiology of copper dyshomeostasis‐associated diseases. Created with BioRender.com.

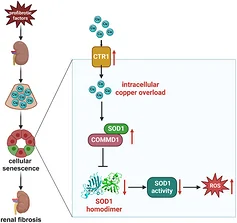

## 1 Introduction

Chronic kidney disease (CKD) is a global health concern, affecting approximately 10% of the adult population worldwide [1]. Recent studies have demonstrated that senescence of renal tubular epithelial cells acts as an initiating event in the progression of renal fibrosis, an inevitable pathway to end‐stage renal disease [2, 3, 4].

Oxidative stress, driven by ROS‐antioxidant imbalance, is a pivotal factor in promoting cell senescence and organ fibrosis [2, 5]. Superoxide dismutase (SOD) is one of the most critical antioxidant enzymes in the body and serves as the core agent for clearing superoxide radicals [6, 7]. SOD1 is the most abundant SOD isoform in the kidney. SOD1 knockout mice display accelerated renal aging and shorten lifespans [8]. Decreased SOD1 activity has been demonstrated to worsen renal injury by enhancing ROS generation in various kidney diseases [9, 10, 11]. However, the precise molecular mechanisms responsible for the reduction of SOD1 activity remain poorly understood.

SOD1 is a copper‐dependent antioxidant enzyme whose catalytic activity strictly depends on copper ions as essential cofactors [12]. The functional SOD1 enzyme exists as a 32‐kDa homodimer that requires multiple maturation steps—copper and zinc incorporation, disulfide bond formation, and homodimerization – to achieve its catalytically active state [13, 14]. Our previous studies and other accumulating evidence have indicated that pathological copper accumulation drives fibrosis in multiple organs, including the lung, liver, and kidney [15, 16, 17, 18]. A cross‐sectional study in elderly populations has revealed a remarkably positive dose‐response association between elevated serum copper levels and increased risk of CKD [19]. Nevertheless, to date, no studies have investigated whether and how intracellular copper accumulation induced by pathological factors impacts SOD1 enzymatic activity.

In the present study, our data demonstrated that intracellular copper overload impaired SOD1 activity in fibrotic kidneys by reducing SOD1 homodimer formation. Lowering copper levels restored SOD1 activity and attenuated ROS accumulation, cell senescence, and fibrosis. We further identified copper metabolism MURR1 domain‐containing 1 (COMMD1) as a copper‐sensitive regulator of SOD1 activity. Mechanistic investigation revealed that copper overload acted upstream to simultaneously upregulate COMMD1 expression and enhance COMMD1‐SOD1 binding affinity, thereby disrupting SOD1 homodimerization and enzymatic activity. These findings uncovered a previously unrecognized copper‐COMMD1‐SOD1 axis that paradoxically suppressed SOD1 activity, redefining the role of copper in SOD1 regulation and providing a potential therapeutic avenue for cell senescence and fibrosis.

## 2 Results

### 2.1 Decreased SOD1 Activity, Intracellular Copper Overload, and Cellular Senescence in Renal Fibrosis

In CKD patients with biopsy‐proven fibrosis, serum SOD1 activity was significantly reduced compared to healthy controls (Figure 1A). Similar decreases were observed in fibrotic kidneys of IRI 28d mice (Figure 1B), UUO 14d mice (Figure 1C), and TGF‐β1‐treated NRK‐52E cells (Figure 1D). To examine whether SOD1 activity impairment promotes renal senescence and fibrosis via ROS accumulation, the SOD mimetic Tempol, which scavenges ROS by mimicking SOD enzymatic function [20], was administered both in vivo and vitro. Our results showed that Tempol treatment significantly alleviated the IRI‐ or TGF‐β1‐induced excessive ROS accumulation, cellular senescence, and renal fibrosis (Figure S1A–G).

**FIGURE 1:**
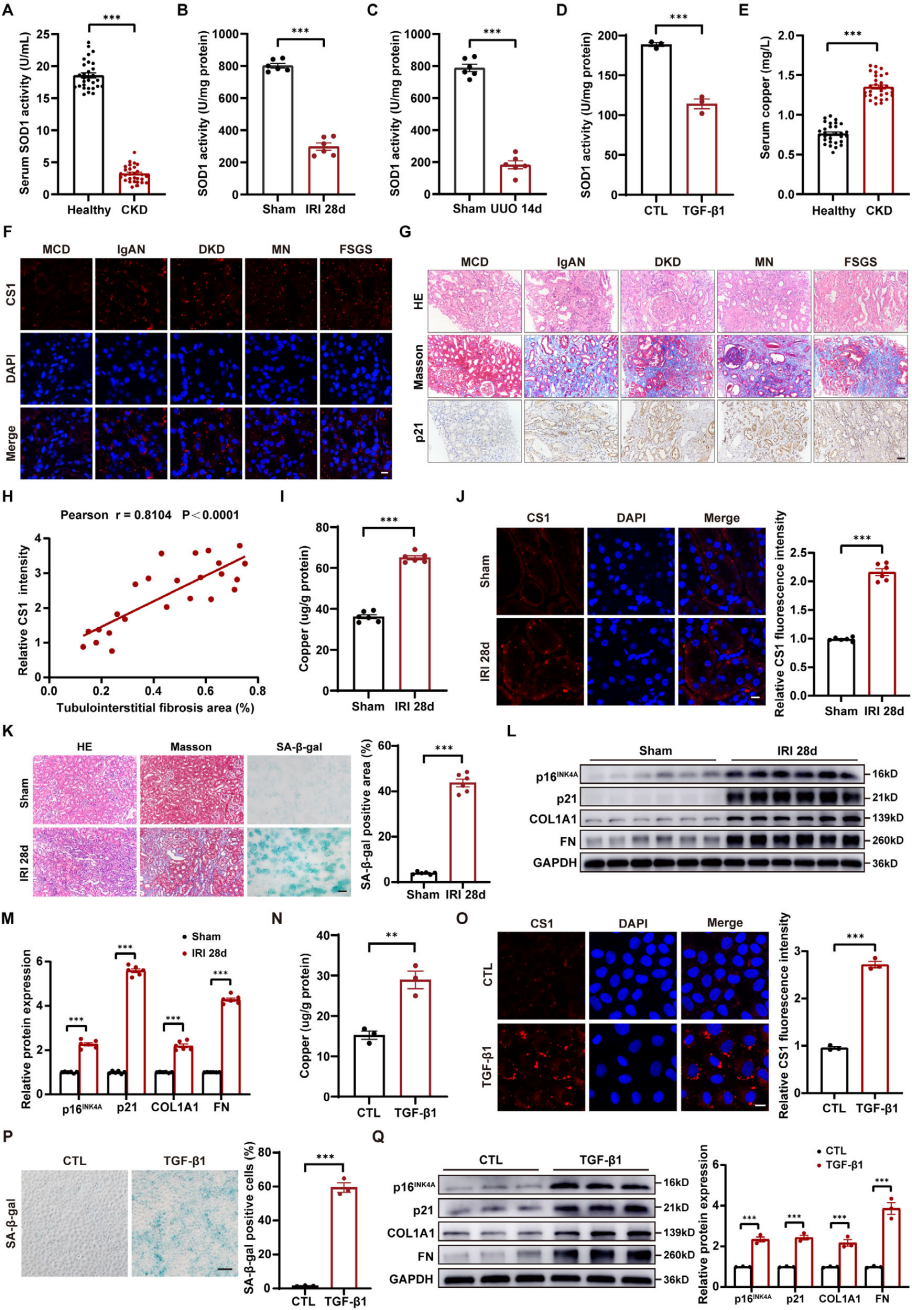
Reduced SOD1 activity, elevated intracellular copper levels, and cellular senescence in renal fibrosis. (A) Serum SOD1 activity was measured in CKD patients with biopsy‐proven fibrosis (n = 30) and healthy volunteers (n = 30). (B) SOD1 activity in the kidney tissues of mice with or without IRI surgery (n = 6). (C) SOD1 activity in the kidney tissues of mice with or without UUO surgery (n = 6). (D) SOD1 activity was assessed in NRK‐52E cells with or without TGF‐β1 treatment (n = 3). (E) Serum copper levels were measured by inductively coupled plasma mass spectrometry (ICP‐MS) in patients with fibrotic CKD (n = 30) and healthy controls (n = 30). (F) Copper content was assessed using Cu^+^‐selective fluorescent probe CS1. Representative CS1 staining images of kidney sections from patients with fibrotic CKD, including IgAN (n = 6), DKD (n = 6), MN (n = 3), and FSGS (n = 3), as well as MCD patients without fibrosis (n = 6). Scale bar = 10 µm. (G) Representative micrographs of p21 immunohistochemistry, HE and Masson's trichrome staining in kidney sections from the same cohort shown in (F). Bar = 50 µm. (H) Linear regression analysis of the correlation between relative CS1 fluorescence intensity and the percentage of fibrotic area in the same cohort shown in (F). Fluorescence intensities were normalized to the mean value of the MCD group. (I) Copper content was detected by ICP‐MS in the kidney tissues of mice with or without IRI surgery (n = 6). (J) Representative images and quantification of CS1 staining in the kidney sections of mice with or without IRI surgery (n = 6). Bar = 10 µm. (K) Representative micrographs of SA‐β‐gal, HE and Masson's trichrome staining in kidney sections of mice with or without IRI surgery. Quantification of SA‐β‐gal positive areas based on SA‐β‐gal staining (n = 6). Bar = 50 µm. (L, M) Western blot images and quantification of p16^INK4A^, p21, COL1A1 and FN in the kidneys of mice with or without IRI surgery (n = 6). (N) Copper content was detected by ICP‐MS in NRK‐52E cells with or without TGF‐β1 treatment (n = 3). (O) Representative images and quantification of CS1 staining in different groups of NRK‐52E cells (n = 3). Bar = 10 µm. (P) Representative micrographs and quantification of SA‐β‐gal staining among different groups. (n = 3). Bar = 50 µm. (Q) Western blot images and quantitative results of p16^INK4A^, p21, COL1A1 and FN across groups. (n = 3). Data were shown as the mean ± SEM. **p < 0.01, ***p < 0.001 versus healthy patients or versus MCD patients or versus Sham group or versus CTL group. Statistical analysis was performed using two‐tailed unpaired Student's t‐test or Mann‐Whitney U test. Correlation analysis was tested with Pearson's correlation.

Furthermore, serum copper levels were significantly elevated in CKD patients compared to healthy controls (Figure 1E). Consistently, Coppersensor‐1 (CS1) staining demonstrated enhanced copper accumulation in fibrotic kidney tissues from patients with multiple CKD etiologies, including IgA nephropathy (IgAN), diabetic kidney disease (DKD), membranous nephropathy (MN), and focal segmental glomerulosclerosis (FSGS), compared with non‐fibrotic minimal change disease (MCD) tissues (Figure 1F). This increase was accompanied by elevated p21 expression detected by immunohistochemical staining, as well as pronounced tubular injury and collagen deposition, as demonstrated by HE and Masson's trichrome staining (Figure 1G). Quantitative analysis further revealed a positive correlation between renal copper accumulation and the extent of tubulointerstitial fibrosis (Figure 1H). Moreover, in the kidney tissues of IRI 28d mice, intracellular copper levels were increased (Figure 1I,J), accompanied by elevated markers of cellular senescence and renal fibrosis (Figure 1K–M). Similar results were also observed in the fibrotic kidneys of UUO 14d mice (Figure S2A–D) and in TGF‐β1‐treated NRK‐52E cells (Figure 1N–Q).

Collectively, these results demonstrated that decreased SOD1 activity promoted ROS accumulation, cellular senescence, and renal fibrosis. Given our previous evidence that intracellular copper overload drives renal senescence and fibrosis [16], these findings suggest that intracellular copper overload may contribute to the reduction of SOD1 activity during renal senescence and fibrosis.

### 2.2 Reducing Intracellular Copper Overload by CTR1 Knockdown Improves SOD1 Activity, Cellular Senescence, and Renal Fibrosis

Subsequently, we further investigated whether intracellular copper overload promoted renal senescence and fibrosis by reducing SOD1 activity. Copper transporter 1 (CTR1) is a copper transport protein, and our previous findings have demonstrated that the activation of profibrotic factor TGF‐β1 signaling increases CTR1 expression, driving enhanced copper influx and intracellular copper accumulation [15]. Thus, in this study, CTR1 knockdown was utilized as a regulatory tool to decrease intracellular copper content. Tubular epithelial cells (TEC)‐specific CTR1 knockout mice (Cre^+^CTR1^fl/fl^) were successfully generated, with genotypes confirmed via PCR (Figure S3A,B). Western blot and IHC analyses together revealed a significant decrease in CTR1 expression in the renal tubular epithelial cells of Cre^+^CTR1^fl/fl^ mice compared to Cre^−^CTR1^fl/fl^ controls (Figure S3C,D).

We established the IRI 28d model in TEC‐specific CTR1 knockout mice (Figure 2A). Compared with IRI 28d‐treated Cre^−^CTR1^fl/fl^ mice, TEC‐specific knockout of CTR1 significantly attenuated renal copper accumulation (Figure 2B) and restored SOD1 activity (Figure 2C). Dihydroethidium (DHE) staining revealed markedly decreased superoxide anion levels in IRI 28d‐treated Cre^+^CTR1^fl/fl^ mice (Figure 2D). Furthermore, TEC‐specific CTR1 knockout alleviated cellular senescence, as indicated by reduced SA‐β‐gal positive areas and decreased protein expressions of senescence markers p16^INK4A^ and p21 (Figure 2E,F). Renal injury and fibrosis were significantly suppressed in IRI 28d‐treated Cre^+^CTR1^fl/fl^ mice compared with IRI 28d‐treated Cre^−^CTR1^fl/fl^ mice, with marked reductions in tubular injury, collagen deposition, and COL1A1 and FN expression (Figure 2E,F).

**FIGURE 2:**
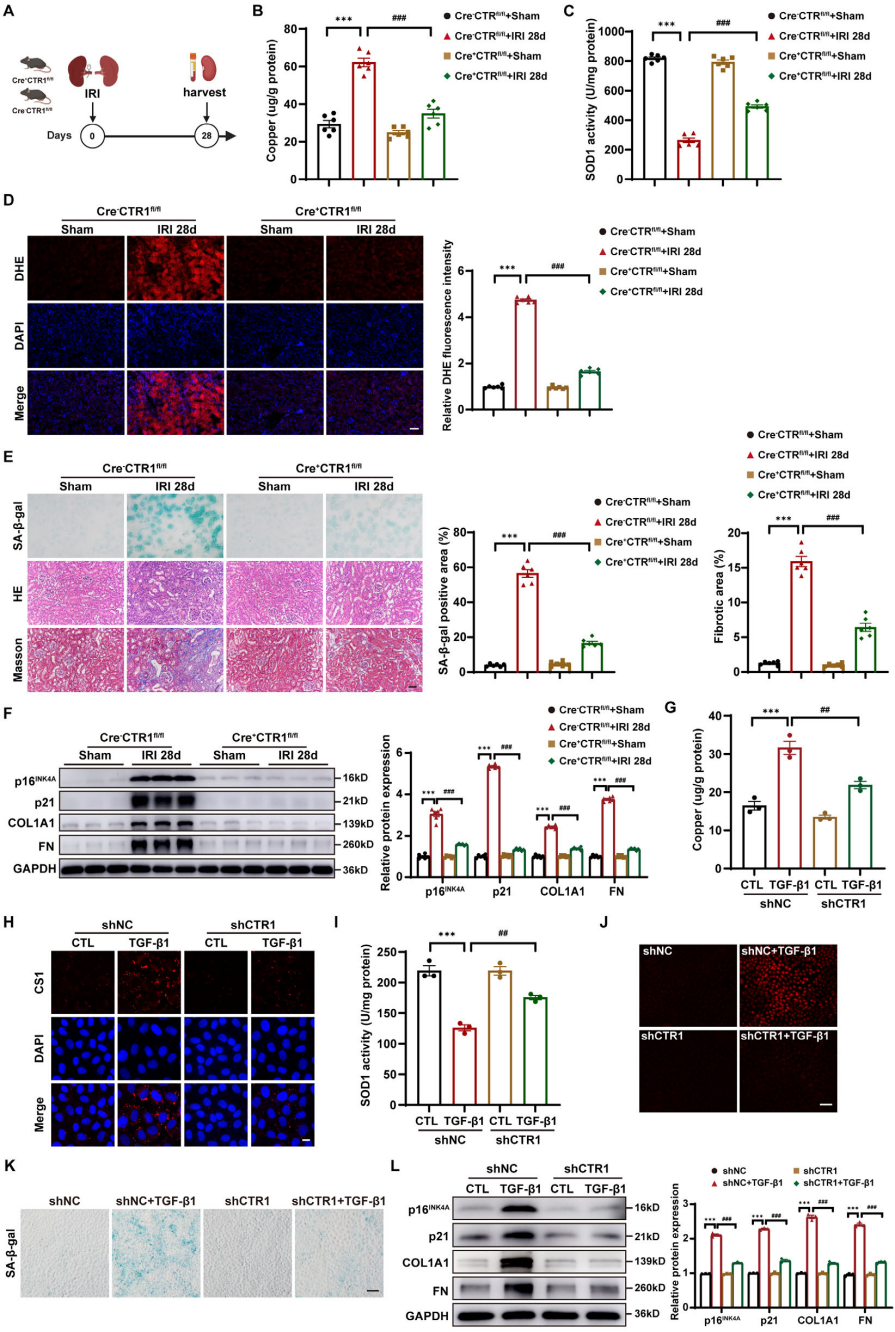
Reducing intracellular copper overload through CTR1 knockdown restores SOD1 activity and prevents cellular senescence and renal fibrosis. (A) Experimental schematic of IRI‐induced renal fibrosis in TEC‐specific CTR1 knockout mice. (B) Copper content was detected by ICP‐MS in the kidneys of Cre^−^CTR1^fl/fl^ and Cre^+^CTR1^fl/fl^ mice with or without IRI surgery (n = 6). (C) SOD1 activity was assessed in the kidneys of Cre^−^CTR1^fl/fl^ and Cre^+^CTR1^fl/fl^ mice with or without IRI surgery (n = 6). (D) Representative DHE staining images and quantification of the average DHE fluorescence intensity relative to the number of nuclei, normalized to the Cre^−^CTR1^fl/fl^+Sham group, across different groups (n = 6). Bar = 100 µm. (E) Representative micrographs of SA‐β‐gal, HE and Masson's trichrome staining in the kidneys from different groups of mice. Quantification of SA‐β‐gal positive areas based on SA‐β‐gal staining and fibrotic areas based on Masson staining (n = 6). Bar = 50 µm. (F) Western blot images and quantification of p16^INK4A^, p21, COL1A1 and FN among different groups (n = 6). (G) Copper content was detected by ICP‐MS in NRK‐52E cells transfected with control shRNA (shNC) or shCTR1 following treatment with or without TGF‐β1 (n = 3). (H) Representative images of CS1 staining among different groups. Bar = 10 µm. (I) SOD1 activity was assessed in NRK‐52E cells across groups (n = 3). (J) Representative DHE images in NRK‐52E cells among different groups (n = 3) (K) Representative images of SA‐β‐gal staining across groups (n = 3). Bar = 50 µm. (L) Western blot images and quantification of p16^INK4A^, p21, COL1A1, and FN in NRK‐52E cells (n = 3). Data were shown as the mean ± SEM. ^***^ *p* < 0.001 versus Cre^−^CTR1^fl/fl^+Sham group or versus shNC group. ##p < 0.01, ###p < 0.001 versus Cre^+^CTR1^fl/fl^+IRI 28d or versus shNC+TGF‐β1 group. Statistical analysis was performed using one‐way ANOVA followed by Tukey's post hoc test.

Similarly, consistent results were observed in TGF‐β1‐stimulated NRK‐52E cells. A stable CTR1 knockdown cell line was generated and validated by Western blot analysis (Figure S3E). CTR1 knockdown alleviated TGF‐β1‐induced effects, as evidenced by reduced copper levels (Figure 2G,H; Figure S3F), restored SOD1 activity (Figure 2I), decreased superoxide anion levels (Figure 2J; Figure S3G), reduced SA‐β‐gal positive cells (Figure 2K; Figure S3H), and suppressed senescence and fibrosis‐associated markers (Figure 2L). Overall, these results indicate that reducing intracellular copper accumulation by CTR1 knockdown attenuates SOD1 activity impairment, ROS overproduction, cellular senescence, and renal fibrosis.

### 2.3 Intracellular Copper Overload Impairs SOD1 Activity by Reducing Homodimer Formation in Renal Fibrosis

We further explored whether intracellular copper overload impaired SOD1 activity at the transcriptional or protein levels. No significant differences in either mRNA or protein levels were observed in IRI 28d‐treated Cre^+^CTR1^fl/fl^ mice compared with IRI 28d‐treated Cre^−^CTR1^fl/fl^ mice (Figure 3A,B). Likewise, no significant differences were observed in NRK‐52E cells co‐treated with CTR1 inhibition and TGF‐β1 compared with cells treated with TGF‐β1 alone (Figure 3C,D). These findings imply that intracellular copper accumulation impairs SOD1 activity independently of its mRNA or protein levels, likely by disrupting its post‐translational maturation process.

**FIGURE 3:**
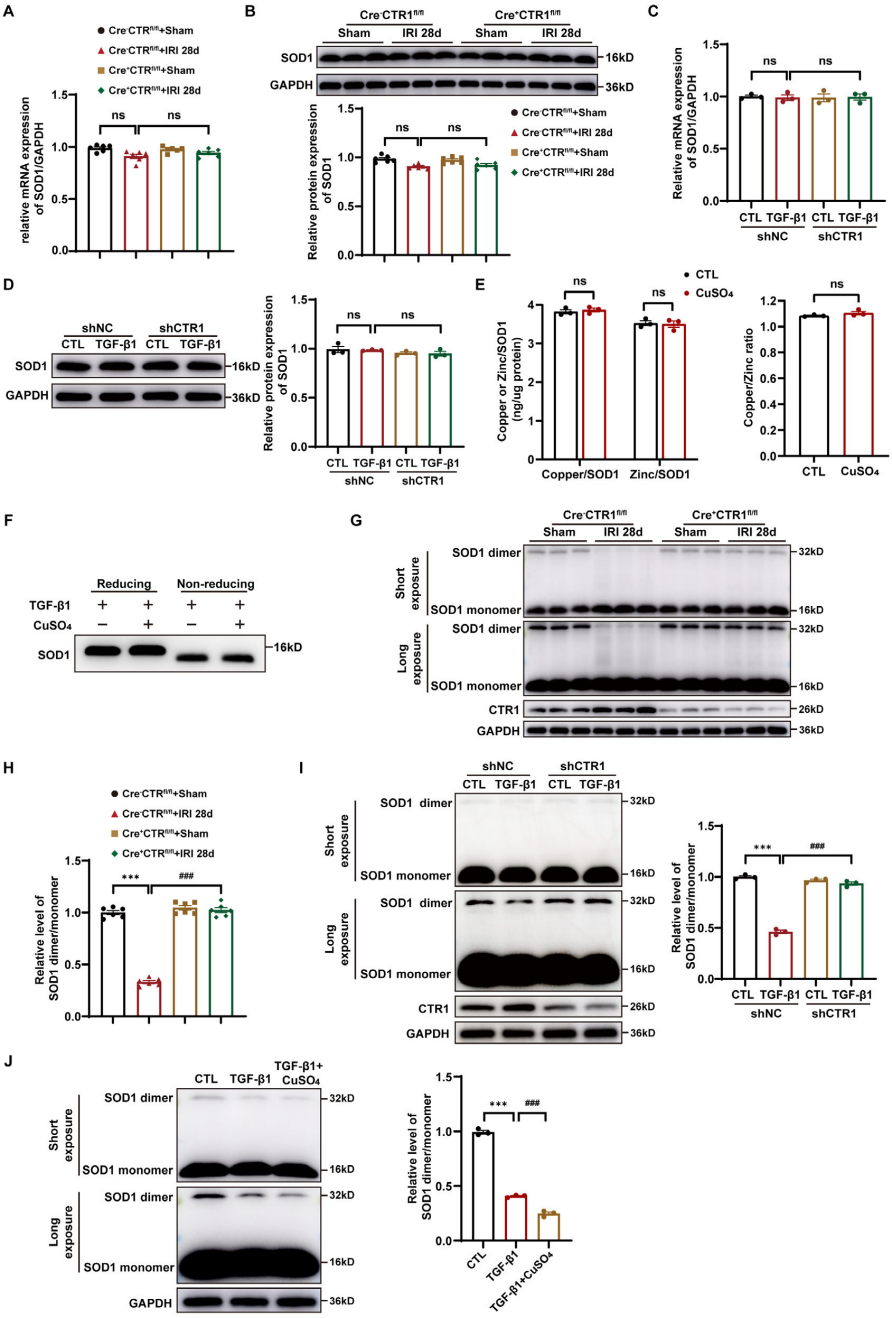
Intracellular copper overload suppresses SOD1 activity by decreasing SOD1 homodimer levels in renal fibrosis. (A) mRNA levels of SOD1 in the kidneys of Cre^−^CTR1^fl/fl^ and Cre^+^CTR1^fl/fl^ mice with or without IRI surgery (n = 6). (B) Western blot images of SOD1 with quantitative analysis in the kidneys of Cre^−^CTR1^fl/fl^ and Cre^+^CTR1^fl/fl^ mice with or without IRI surgery (n = 6). (C) mRNA levels of SOD1 in NRK‐52E cells transfected with shNC or shCTR1, followed by treatment with or without TGF‐β1 (n = 3). (D) Western blot images of SOD1 with quantitative analysis in NRK‐52E cells transfected with shNC or shCTR1, followed by treatment with or without TGF‐β1 (n = 3). (E) ICP‐MS quantification of Cu and Zn contents and Cu/Zn ratio in recombinant human SOD1 with or without CuSO_4_ incubation following desalting (n = 3). (F) SOD1 migration under reducing and non‐reducing SDS‐PAGE in TGF‐β1‐treated NRK‐52E cells with or without CuSO_4_ treatment (n = 3). (G, H) Western blot images and quantification of SOD1 monomers and dimers in the kidneys of Cre^−^CTR1^fl/fl^ and Cre^+^CTR1^fl/fl^ mice with or without IRI surgery (n = 6). (I) Western blot images and quantification of SOD1 monomers and dimers in NRK‐52E cells transfected with shNC or shCTR1 and then treated with or without TGF‐β1 (n = 3). (J) Western blot images and quantification of SOD1 monomers and dimers in TGF‐β1‐treated NRK‐52E cells with or without CuSO_4_ stimulation (n = 3). Data were shown as the mean ± SEM. ^***^p < 0.001 versus Cre^−^CTR1^fl/fl^+Sham group or versus shNC group. ###p < 0.001 versus Cre^+^CTR1^fl/fl^+IRI 28d or versus shNC+TGF‐β1 group. Statistical analysis was performed using one‐way ANOVA followed by Tukey's post hoc test or two‐tailed unpaired Student's t‐test.

SOD1 maturation requires copper/zinc insertion, disulfide oxidation, and homodimer formation – the final and essential step for its activation [12, 21]. To determine whether altered metal occupancy contributed to SOD1 inactivation under copper overload conditions, Cu and Zn contents in recombinant human SOD1 were quantified by ICP‐MS following CuSO_4_ incubation and desalting. No significant differences were observed in SOD1‐associated copper content, zinc content, or the Cu/Zn ratio between control and CuSO_4_‐incubated samples (Figure 3E), suggesting that copper overload did not alter SOD1 metal occupancy. We next examined whether copper overload affected the intramolecular disulfide bond status of SOD1 using reducing and non‐reducing SDS‐PAGE in TGF‐β1‐treated NRK‐52E cells with or without CuSO_4_ treatment. In both groups, SOD1 showed faster migration under non‐reducing conditions than under reducing conditions (Figure 3F), indicating the presence of oxidized intramolecular disulfide bonds. Importantly, CuSO_4_ treatment did not alter the electrophoretic mobility of SOD1 under non‐reducing conditions (Figure 3F), suggesting that copper overload did not significantly affect the SOD1 intramolecular disulfide oxidation status.

To determine the role of intracellular copper overload in regulating SOD1 homodimer levels during renal fibrosis, we evaluated SOD1 homodimer levels following CTR1 knockdown in vivo and in vitro. In vivo, SOD1 homodimers levels were significantly decreased in the Cre^−^CTR1^fl/fl^ mice treated with IRI 28d, which was reversed by lowering copper levels in Cre^+^CTR1^fl/fl^ mice treated with IRI 28d (Figure 3G,H). In NRK‐52E cells, SOD1 homodimer levels were markedly reduced by TGF‐β1 treatment, an effect that was significantly attenuated by reducing copper content through CTR1 inhibition (Figure 3I) and aggravated by CuSO_4_ supplementation (Figure 3J). Taken together, these results suggest that intracellular copper overload impairs SOD1 activity in renal fibrosis primarily by reducing homodimer formation, independent of altered Cu/Zn metalation or impaired intramolecular disulfide bond formation.

### 2.4 COMMD1 Upregulation Inhibits SOD1 Homodimerization and Enzymatic Activity

To identify potential mediators through which intracellular copper overload impaired SOD1 homodimer formation, we performed DIA‐based proteomic analysis in control and CuSO_4_‐treated NRK‐52E cells. PCA revealed clear group separation with good reproducibility, and 46 differentially expressed proteins were identified and visualized by volcano plot and heatmap (Figure S4A–C). To further focus on SOD1‐associated regulatory mechanisms, we next constructed a SOD1‐centered interaction network using STRING and BioGRID databases, defining 15 candidate interacting proteins (Figure S5). Integration of the DIA proteomic dataset with this interaction network revealed that 12 of the 15 candidate proteins were detected. Notably, among the detected proteins, COMMD1 was significantly upregulated in CuSO_4_‐treated cells (log2FC > 1, false discovery rate < 0.05), while no significant changes were observed in the remaining candidates (Figure 4A,B; Table S1).

**FIGURE 4:**
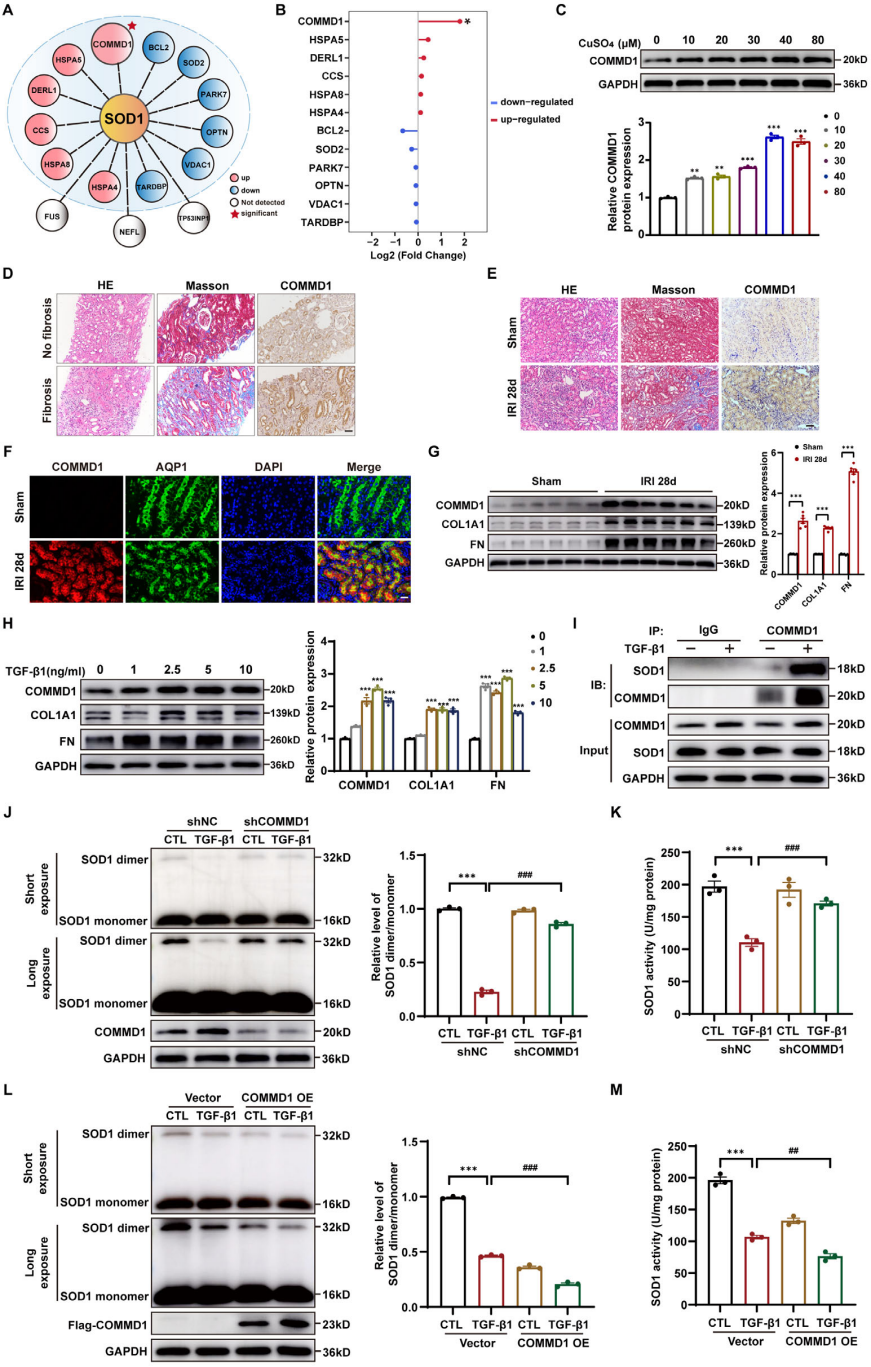
COMMD1 elevation impairs SOD1 homodimerization and enzymatic activity. (A) Schematic illustration of SOD1‐centered interaction landscape integrated with DIA proteomics. Node colors indicate differential expression (red, upregulated; blue, downregulated; gray, not detected). Red star indicates statistically significant changes. Edges represent protein‐protein interactions. (B) Lollipop plot showing log2FC values of quantified SOD1‐associated proteins identified in the DIA dataset. * indicates |log2 FC| ≥ 1 and FDR < 0.05. (C) Western blot images and quantification of COMMD1 in NRK52E cells treated with different doses of CuSO_4_ (n = 3). (D) Representative micrographs of COMMD1 immunohistochemistry, HE and Masson's trichrome staining in the kidney sections from IgAN patients with fibrosis and MCD patients with no fibrosis (n = 3). Bar = 50 µm. (E) Representative micrographs of COMMD1 immunohistochemistry, HE, Masson's trichrome staining micrographs in the kidney sections of mice with or without IRI surgery (n = 3). Bar = 50 µm. (F) Representative immunofluorescence micrographs of COMMD1 in kidney sections from mice with or without IRI surgery (n = 3). Bar = 25 µm. (G) Western blot images and quantification of COMMD1, COL1A1 and FN in the kidneys of mice with or without IRI surgery (n = 6). (H) Western blot images and quantification of COMMD1, COL1A1 and FN in NRK52E cells treated with different doses of TGF‐β1 (n = 3). (I) Co‐IP of COMMD1 and SOD1 in NRK‐52E cells with or without TGF‐β1 stimulation for 48 h. Lysates were immunoprecipitated with control IgG or COMMD1 antibodies and then analyzed by Western blot with the indicated antibody (n = 3). (J) Western blot images and quantitative analysis of SOD1 monomers and dimers in COMMD1‐knockdown NRK‐52E cells treated with or without TGF‐β1 (n = 3). (K) SOD1 activity was assessed in NRK‐52E cells among different groups (n = 3). (L) Western blot images and quantitative analysis of SOD1 monomers and dimers in COMMD1‐overexpressing NRK‐52E cells treated with or without TGF‐β1 (n = 3). (M) SOD1 activity was assessed across groups (n = 3). Data were shown as the mean ± SEM. **p < 0.01, ^***^p < 0.001 versus CTL group or versus Sham group or versus shNC group or versus Vector group. ##p < 0.01, ###p < 0.001 versus shNC+TGF‐β1 group or versus Vector+TGF‐β1 group. Statistical analysis was performed using two‐tailed unpaired Student's t‐test or one‐way ANOVA followed by Tukey's post hoc test.

Consistently, COMMD1 protein upregulation was validated in NRK‐52E cells treated with different concentrations of CuSO_4_ (Figure 4C). COMMD1 mRNA levels also increased with CuSO_4_ treatment and reversed by the copper chelator tetrathiomolybdate (Figure S6A,B). To assess whether copper affected COMMD1 protein stability, cycloheximide (CHX) chase assays were performed in control and CuSO_4_‐treated cells. No significant difference in COMMD1 protein degradation was observed between the two groups (Figure S6C). Immunohistochemistry or immunofluorescence analysis revealed marked COMMD1 upregulation in fibrotic kidneys from IgAN patients (Figure 4D) and IRI 28d mice (Figure 4E,F), predominantly localized to TECs. Western blot analysis corroborated these findings (Figure 4G). Similar increases were observed in both UUO models (Figure S7A–C) and TGF‐β1‐treated NRK‐52E cells (Figure 4H; Figure S7D). Moreover, Co‐IP analysis demonstrated a physical interaction between COMMD1 and SOD1, which was enhanced upon TGF‐β1 stimulation (Figure 4I).

To validate the role of COMMD1 in regulating SOD1 dimerization and enzymatic activity, stable NRK‐52E cell lines with COMMD1 knockdown or overexpression were generated and confirmed (Figure S8A,B). COMMD1 knockdown attenuated TGF‐β1‐induced reductions in SOD1 homodimer levels and enzymatic activity (Figure 4J,K), whereas COMMD1 overexpression further aggravated these effects (Figure 4L,M). COMMD1 has been reported to facilitate the ubiquitination and subsequent proteasomal degradation of its interacting proteins [22, 23]. To investigate whether a similar mechanism contributes to SOD1 regulation, NRK‐52E cells were transfected with vector or COMMD1 overexpression plasmids and treated with or without the proteasome inhibitor MG132. Western blot analysis showed that COMMD1 overexpression did not significantly alter total SOD1 protein abundance under either condition (Figure S9). Together, these data suggest that COMMD1 upregulation interacts with SOD1 and suppresses SOD1 homodimerization and enzymatic activity independently of proteasomal degradation.

### 2.5 Intracellular Copper Overload Facilitates COMMD1‐SOD1 Interaction, Disrupting SOD1‐SOD1 Dimerization and Enzymatic Activity

We next performed a series of experiments to elucidate the role of copper overload in COMMD1‐mediated disruption of SOD1 homodimerization. Co‐IP analysis showed that CTR1 knockdown markedly weakened the COMMD1‐SOD1 interaction compared with TGF‐β1 treatment alone (Figure 5A). Conversely, CuSO_4_ supplementation significantly enhanced this interaction in TGF‐β1‐treated NRK‐52E cells (Figure 5B). Importantly, CTR1 knockdown attenuated the TGF‐β1‐induced upregulation of COMMD1 protein expression (Figure 5C). These data suggest that copper overload promotes COMMD1 upregulation and facilitates the COMMD1‐SOD1 interaction.

**FIGURE 5:**
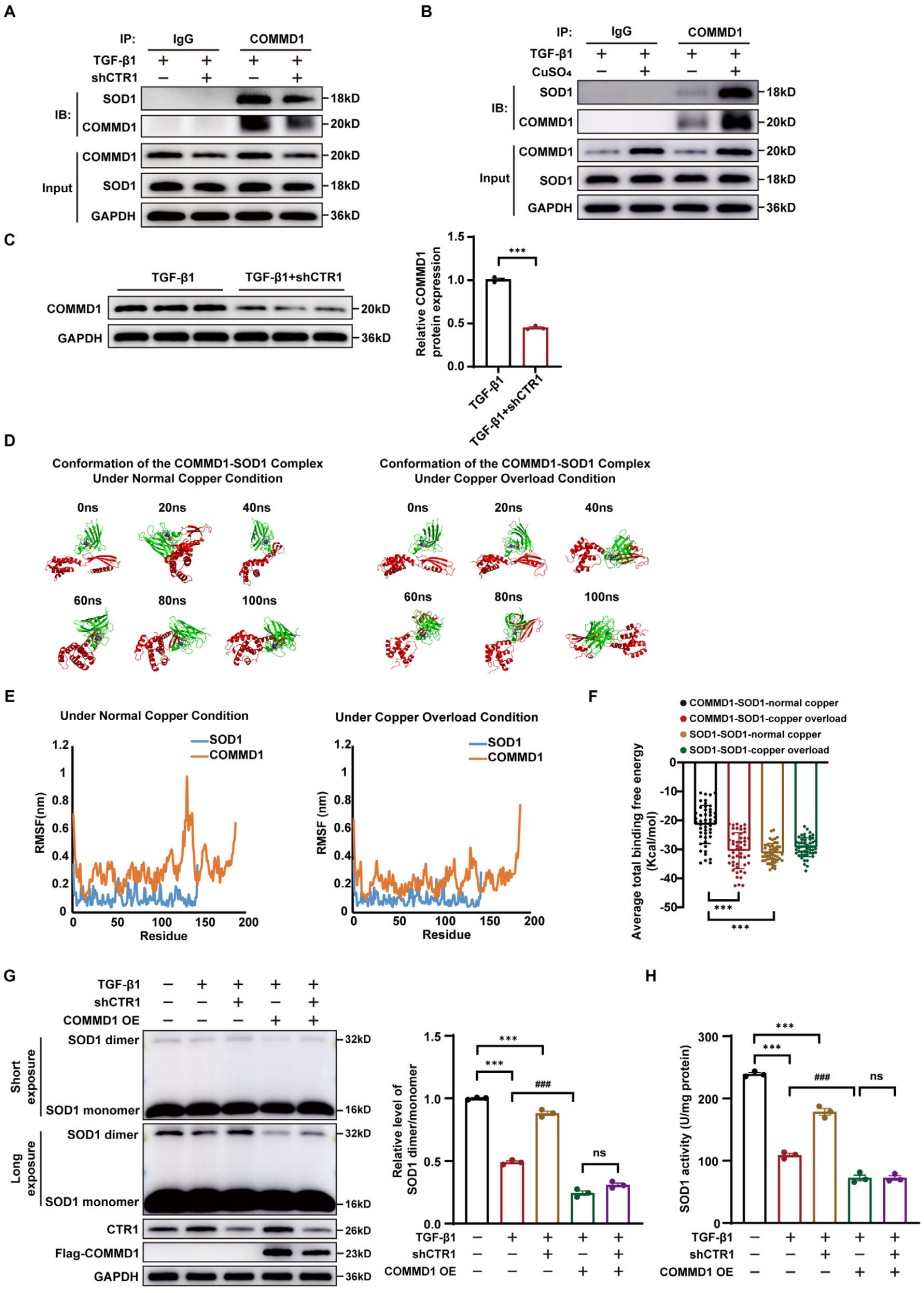
Elevated intracellular copper enhances COMMD1‐SOD1 binding, leading to impaired SOD1 homodimerization and enzymatic activity. (A) Co‐IP of COMMD1 and SOD1 in TGF‐β1‐treated NRK‐52E cells with or without shCTR1 knockdown (n = 3). (B) Co‐IP of COMMD1 and SOD1 in TGF‐β1‐treated NRK‐52E cells with or without CuSO_4_ stimulation (n = 3). (C) Western blot images and quantitative results of COMMD1 in TGF‐β1‐ treated NRK52E cells with or without CuSO_4_ administration (n = 3). (D) The overall three‐dimensional conformations of the COMMD1‐SOD1 complex under different molecular dynamics simulation systems were evaluated from the same perspective at six time points (0, 20, 40, 60, 80, and 100 ns). The red color represents COMMD1, and the green color represents SOD1. (E) The RMSF of all amino acid residues in COMMD1‐SOD1 complex was assessed under different molecular dynamics simulation systems. (F) The MM/GBSA method was employed to calculate the average total binding free energies under four molecular dynamics simulation systems (n = 49). (G) Western blot images and quantitative results of SOD1 monomers and dimers in different groups of NRK‐52E cells (n = 3). (H) SOD1 activity was measured in different groups of NRK‐52E cells (n = 3). Data were shown as the mean ± SEM. ^***^p < 0.001 versus TGF‐β1 group or versus COMMD1‐SOD1‐normal copper group or versus shNC group. ###p < 0.001 versus shNC+TGF‐β1 group. Statistical analysis was performed using two‐tailed unpaired Student's t‐test or one‐way ANOVA followed by Tukey's post hoc test.

To gain further mechanistic insight, molecular dynamics (MD) simulations were performed to assess the impact of copper levels on COMMD1‐SOD1 and SOD1‐SOD1 interactions under normal and copper overload conditions. The system remained stable throughout the simulations (Figure S10A), with no notable changes in the overall three‐dimensional conformation of either complex (Figure 5D ； Figure S10B) or in the secondary structure of COMMD1 and SOD1 (Figure S10C).

Notably, significant findings were observed in Root mean square fluctuation (RMSF) and MM/GBSA binding free energy analyses. Higher RMSF fluctuations reflect greater residue flexibility and complex instability, whereas lower fluctuations indicate enhanced structural stability. Under normal copper conditions, elevated RMSF values around residue 130 of COMMD1 suggested local structural instability in the COMMD1‐SOD1 complex. In contrast, this region remained stable under copper overload, suggesting that copper overload may stabilize COMMD1 and promote its binding to SOD1 (Figure 5E). Moreover, the SOD1‐SOD1 complex remained stable regardless of copper levels (Figure S10D). To further validate these results, MM/GBSA‐based binding free energy calculations were conducted using 49 frames extracted from the stabilized MD trajectory. Under normal copper conditions, the COMMD1‐SOD1 complex exhibited a greater absolute binding free energy than the SOD1‐SOD1 complex. However, under copper overload, its binding affinity significantly increased, slightly exceeding that of the SOD1‐SOD1 complex (Figure 5F). Collectively, these findings suggest that copper overload stabilizes the local conformation of COMMD1 and enhances COMMD1‐SOD1 binding, potentially inhibiting SOD1‐SOD1 interaction and disrupting SOD1 homodimerization.

To further elucidate whether copper acted upstream of COMMD1 in regulating SOD1, we designed the relevant experimental groups. In COMMD1‐overexpressing cells with TGF‐β1 treatment, reducing copper content via CTR1 knockdown failed to rescue SOD1 homodimer levels (Figure 5G) or its enzymatic activity (Figure 5H). Overall, these findings indicate that copper overload acts as upstream of COMMD1, enhancing COMMD1‐SOD1 interaction by simultaneously upregulating COMMD1 and increasing COMMD1‐SOD1 binding affinity, thereby suppressing SOD1 homodimerization and enzymatic activity.

### 2.6 COMMD1 Promotes TGF‐β1‐Induced ROS Accumulation, Cellular Senescence, and ECM Production

To assess the role of COMMD1 in ROS accumulation and cellular senescence under renal fibrotic conditions, stable NRK‐52E cell lines with COMMD1 knockdown or overexpression were treated with TGF‐β1 in vitro. Our results showed that COMMD1 knockdown significantly alleviated the TGF‐β1‐induced excessive ROS accumulation, as evidenced by lower superoxide anion levels (Figure 6A). In addition, COMMD1 knockdown also markedly attenuated TGF‐β1‐induced cellular senescence, as indicated by fewer SA‐β‐gal positive cells (Figure 6B) and reduced protein expressions of p16^INK4A^ and p21 (Figure 6C). Furthermore, COMMD1 inhibition significantly suppressed TGF‐β1‐induced upregulation of the fibrotic markers COL1A1 and FN (Figure 6C). In contrast, COMMD1 overexpression aggravated TGF‐β1‐induced excessive ROS accumulation and enhanced cellular senescence and ECM production (Figure 6D–F). Together, these findings suggest that COMMD1 regulates ROS accumulation and subsequent cellular senescence and ECM deposition, highlighting its pivotal role in renal fibrotic responses.

**FIGURE 6:**
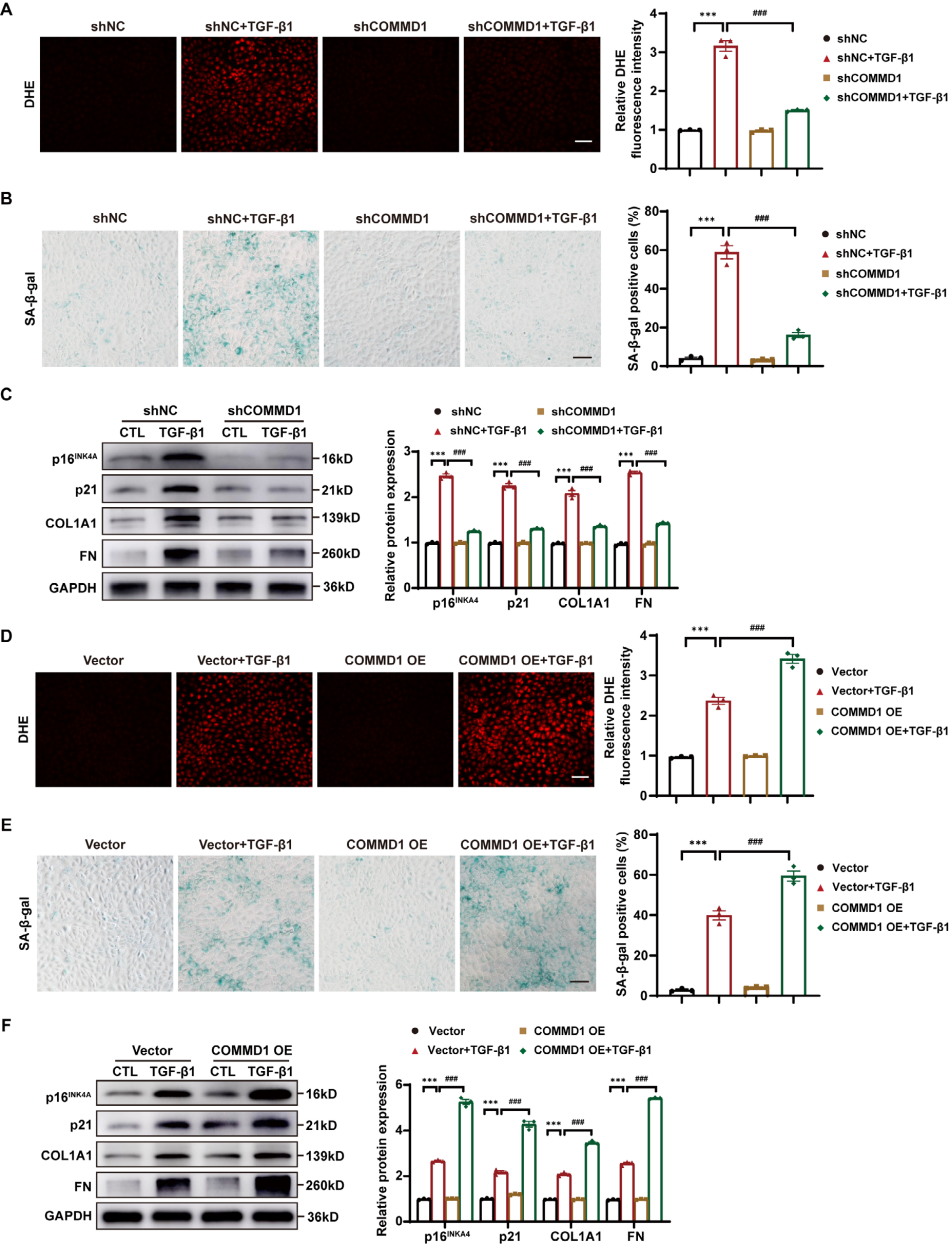
COMMD1 facilitates TGF‐β1‐induced ROS accumulation, cellular senescence, and ECM deposition. (A) Representative DHE images and quantification of the average DHE fluorescence intensity, normalized to shNC group, in COMMD1‐knockdown NRK‐52E cells with or without TGF‐β1 treatment (n = 3). Bar = 50 µm. (B) Representative images and quantification of SA‐β‐gal staining in different groups of NRK‐52E cells (n = 3). Bar = 50 µm. (C) Western blot images and quantitative analysis of p16^INK4A^, p21, COL1A1 and FN in COMMD1‐knockdown NRK‐52E cells treated with or without TGF‐β1 (n = 3). (D) Representative DHE images and quantitative analysis in COMMD1‐overexpressing NRK‐52E cells treated with or without TGF‐β1 (n = 3). Bar = 50 µm. (E) Representative images and quantification of SA‐β‐gal staining among different groups (n = 3). Bar = 50 µm. (F) Western blot images and quantitative analysis of p16^INK4A^, p21, COL1A1 and FN across groups (n = 3). Data were shown as the mean ± SEM. ***p < 0.001 versus shNC group or versus Vector group. ###p < 0.001 versus shNC+TGF‐β1 group or versus Vector+TGF‐β1 group. Statistical analysis was performed using one‐way ANOVA followed by Tukey's post hoc test.

### 2.7 COMMD1 Downregulation Restores SOD1 Homodimerization and Activity to Ameliorate IRI‐induced ROS Accumulation, Cellular Senescence, and Renal Fibrosis

To further validate the effects of COMMD1 in vivo, we achieved effective COMMD1 depletion in the kidney of C57BL/6J mice via AAV9‐shCOMMD1 administration, as confirmed by markedly reduced COMMD1 mRNA and protein levels (Figure S11A,B). The IRI 28d model was established in mice with or without COMMD1 depletion (Figure 7A). Our results revealed that COMMD1 downregulation mitigated IRI‐induced decrease in SOD1 homodimer levels (Figure 7B) and restored SOD1 enzymatic activity (Figure 7C), thereby markedly reducing superoxide anion accumulation (Figure 7D). Moreover, COMMD1 downregulation significantly attenuated cellular senescence, as evidenced by decreased SA‐β‐gal positive areas (Figure 7E) and reduced expressions of senescence‐associated proteins p16^INK4A^ and p21 (Figure 7F). Additionally, COMMD1 inhibition substantially alleviated kidney fibrosis, as demonstrated by reduced tubular injury, decreased interstitial ECM deposition (Figure 7E), and lower protein levels of fibrotic markers COL1A1 and FN (Figure 7F). Altogether, these results confirm the essential role of COMMD1 in impairing SOD1 activation, thereby amplifying ROS accumulation and promoting renal senescence and fibrosis.

**FIGURE 7:**
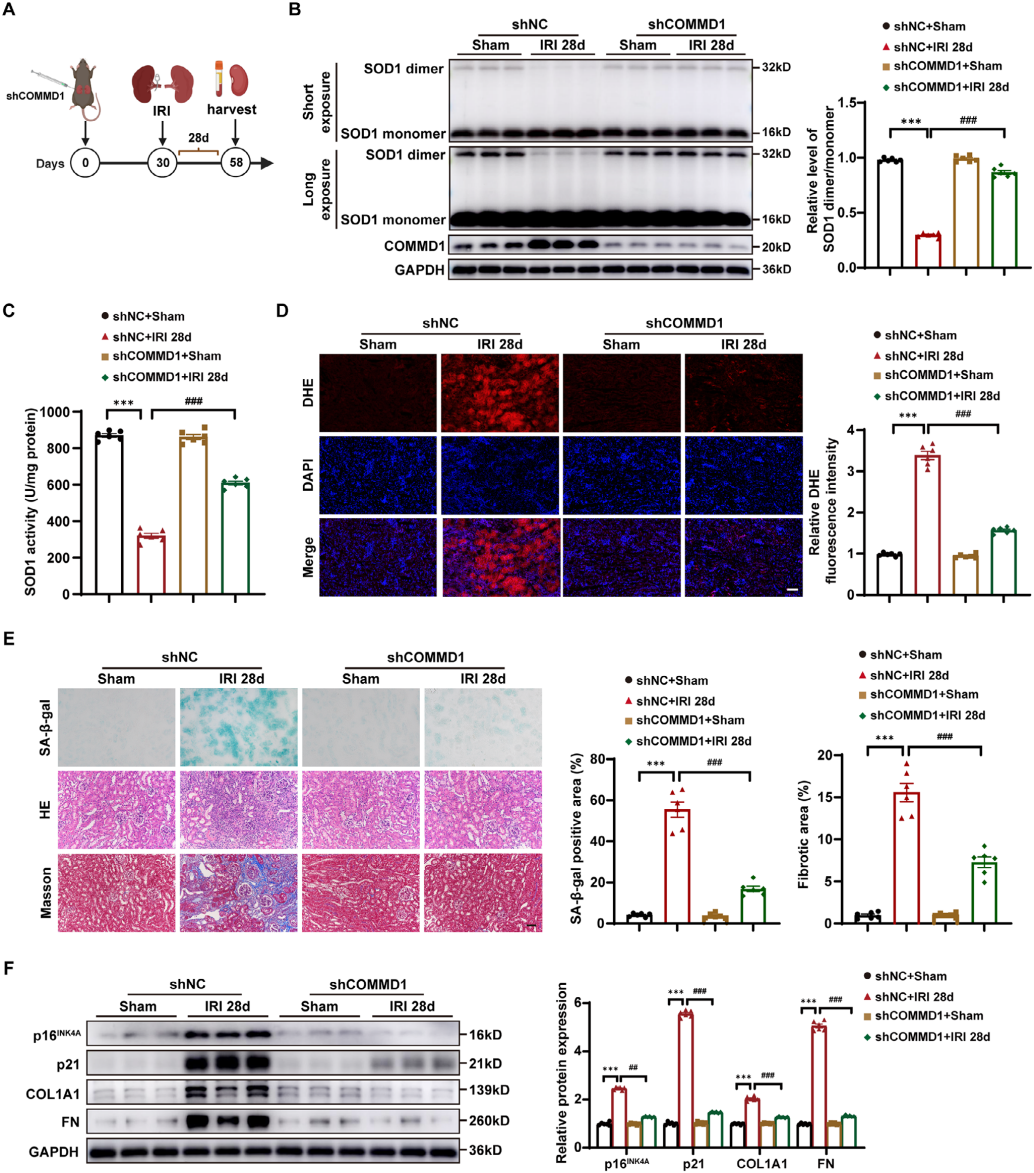
COMMD1 downregulation improves SOD1 homodimerization and enzymatic activity, attenuating IRI‐induced ROS accumulation, cellular senescence and renal fibrosis. (A) Experimental schematic of intrarenal AAV9‐shCOMMD1 administration followed by IRI‐induced renal fibrosis in mice. (B) Western blot images and quantification of SOD1 monomers and dimers in the kidneys of shNC‐ and shCOMMD1‐transfected mice with or without IRI surgery (n = 6). (C) SOD1 activity was assessed among different groups (n = 6). (D) Representative DHE staining images and quantification in the kidneys from different groups of mice (n = 6). Bar = 100 µm. (E) Representative micrographs of SA‐β‐gal staining, HE and Masson's trichrome staining in kidney sections from shNC‐ and shCOMMD1‐transfected mice with or without IRI surgery. Quantification of SA‐β‐gal positive areas based on SA‐β‐gal staining and fibrotic areas based on Masson's trichrome staining (n = 6). Bar = 50 µm. (F) Western blot images with quantitative analysis of p16^INK4A^, p21, COL1A1 and FN in the kidneys from the indicated groups of mice (n = 6). Data were shown as the mean ± SEM. ***p < 0.001 versus shNC+Sham group. ###p < 0.001 versus shNC+IRI 28d group. Statistical analysis was performed using one‐way ANOVA followed by Tukey's post hoc test.

## 3 Discussion

This study demonstrated for the first time that intracellular copper overload facilitated COMMD1‐mediated suppression of SOD1 activity, resulting in increased ROS accumulation and promoting cell senescence and renal fibrosis. Mechanistically, copper overload enhanced COMMD1‐SOD1 interaction by concurrently upregulating COMMD1 and increasing the binding affinity of COMMD1‐SOD1, disrupting SOD1 homodimerization and enzymatic function. We proposed a general copper‐COMMD1‐SOD1 regulatory axis that redefined how copper overload suppressed antioxidant defenses under pathological conditions.

Although it was widely recognized that SOD1 requires copper ions as indispensable cofactors for its antioxidant function [12, 13], our study demonstrated that intracellular copper overload played a pivotal role in driving the decline in SOD1 activity, resulting in excessive ROS accumulation, accelerated cell senescence, and renal fibrosis progression. Our study provided the first experimental evidence that SOD1's copper‐dependent activity was context‐dependent, exhibiting opposing regulatory patterns under physiological versus pathological copper overload conditions. This copper‐induced SOD1 inhibitory checkpoint refines and extends the conceptual framework linking copper homeostasis to oxidative stress.

As a 32‐kDa homodimer, mature SOD1 requires intricate post‐translational modifications – including copper and zinc incorporation, disulfide bond formation, and homodimerization – for its functional activation [12, 21]. Our data demonstrated that intracellular copper accumulation under fibrotic conditions impaired SOD1 activity predominantly by disrupting homodimer formation, without affecting SOD1 mRNA expression, total protein levels, Cu/Zn metal occupancy, or intramolecular disulfide oxidation. These findings suggest that copper overload selectively disrupts the final maturation step of SOD1. In amyotrophic lateral sclerosis (ALS), mutations in SOD1 drive protein misfolding and disrupt homodimerization, leading to monomerization, aberrant interactions, and proteotoxic aggregation [24, 25] – a pathological cascade underscoring the critical role of proper homodimerization in SOD1 activation. In contrast to ALS‐associated genetic mutations, our findings uncovered a distinct non‐genetic pathological mechanism that converges, at least in part, on a shared outcome: disruption of SOD1 homodimerization.

COMMD1, a member of the COMMD protein family, serves as a multifunctional regulator of copper homeostasis, NF‐κB activity, HIF‐1α signaling, cell cycle arrest, and sodium transport [26, 27, 28]. While an earlier molecular biology study has reported that COMMD1 negatively regulates SOD1 homodimer formation [29], our study provided the first pathological evidence linking this regulatory mechanism to cell senescence and fibrosis. We observed that COMMD1 was upregulated under fibrotic conditions, and its interaction with SOD1 was markedly enhanced by TGF‐β1 treatment. Notably, COMMD1 overexpression suppressed SOD1 homodimerization and enzymatic activity without altering total SOD1 protein levels, which remained stable even upon MG132 treatment. This outcome effectively excludes the involvement of proteasomal degradation‐dependent mechanism, a well‐established role of COMMD1 [22, 23]. Conversely, COMMD1 knockdown restored SOD1 homodimerization and activity, thereby ameliorating ROS accumulation, cellular senescence, and renal fibrosis. Our findings established COMMD1 as a novel endogenous inhibitor of SOD1 activity by suppressing its homodimerization. COMMD1 may serve as a pivotal molecular switch modulating SOD1 activation in aging and fibrosis‐associated diseases.

Another fascinating discovery of this study was the identification of a novel regulatory mechanism whereby copper overload acted as an upstream regulator of COMMD1‐mediated inhibition of SOD1 homodimerization and activation. Co‐IP analysis demonstrated that copper overload enhanced COMMD1‐SOD1 interaction. Mechanistically, our findings suggest that intracellular copper overload functions as a copper‐responsive regulator of COMMD1 upregulation. In addition, a previous study has reported that under basal copper conditions, copper insertion and disulfide oxidation induce conformational changes in SOD1 subunits, thereby facilitating homodimer formation [30]. However, to date, the structural effects of elevated copper levels on SOD1‐SOD1 and COMMD1‐SOD1 complexes remain unexplored. Through comprehensive molecular dynamics simulations, we had several key observations: although copper overload did not significantly affect the overall three‐dimensional structural stability of either the SOD1‐SOD1 or COMMD1‐SOD1 complexes, it triggered local conformational changes and altered the binding affinity of the COMMD1‐SOD1 complex. Notably, under copper overload conditions, the binding affinity of COMMD1‐SOD1 complex increased and slightly exceeded that of SOD1 homodimers. These computational results proposed a structural competition model in which copper overload preferentially stabilized the COMMD1‐SOD1 interaction, disrupting SOD1 homodimer assembly. Further biochemical analyses confirmed that copper overload acted upstream to upregulate COMMD1 and enhance COMMD1‐SOD1 binding, ultimately inhibiting SOD1 homodimerization and enzymatic activity.

Though our study demonstrates that copper overload increases COMMD1 expression, the upstream mechanism underlying COMMD1 upregulation remains unclear. Our findings suggest that copper‐induced COMMD1 upregulation is mainly driven by increased COMMD1 mRNA expression rather than altered protein stability, implicating transcriptional regulation. Antioxidant 1 copper chaperone (ATOX1), a copper‐dependent transcription factor, may potentially contribute to this process, as copper stimulation has been reported to promote its nuclear translocation and transcriptional activity [31, 32, 33, 34]. However, this possibility remains speculative, and definitive validation of this proposed mechanism would require extensive mechanistic investigation in future studies.

In conclusion, we have uncovered a previously unrecognized mechanism linking copper homeostasis to oxidative stress in cell senescence and fibrosis, and propose a general copper‐COMMD1‐SOD1 regulatory axis that redefines our understanding of how pathological copper overload suppresses antioxidant defenses. These findings provide important conceptual and translational insights for the development of targeted therapeutic strategies against senescence and fibrosis.

## 4 Experimental Section

### 4.1 Human Kidney Tissues

The human kidney tissues and serum samples were obtained from the Department of Nephrology, Tongji Hospital, Shanghai, China. Control tissues were collected from patients diagnosed with biopsy‐proven MCD without fibrosis (n = 6). Fibrotic kidney tissues were acquired from multiple CKD etiologies exhibiting characteristic fibrotic pathology, including IgAN (n = 6), DKD (n = 6), MN (n = 3), and FSGS (n = 3). Human serum samples were collected from healthy volunteers (n = 30) and CKD patients with biopsy‐proven fibrosis (n = 30). All specimens were collected with approval from the Human Subjects Committee of Tongji Hospital (Approval No. K‐W‐2021‐012) and written informed consent voluntarily provided by all participants.

### 4.2 Generation of Tubular Epithelial Cells (TEC)‐Specific CTR1 Knockout Mice

To generate the CTR1^flox/flox^ (referred to as CTR1^fl/fl^) inbred mouse line, the targeting vector was inserted into two loxP sites on the flank of the second and third exons of CTR1 in the zygotes of C57BL/6J mice. CTR1^fl/fl^ mice were generated by GemPharmatech (Jiangsu, China). These mice were subsequently crossed with Ggt1‐Cre mice (which express Cre under the regulation of the Ggt1 promoter; Jackson, https://www.jax.org/strain/012841) to produce TEC‐specific CTR1 knockout mice (Cre^+^CTR1^fl/fl^). Littermates of CTR1^fl/fl^ mice lacking Cre expression were used as controls (Cre^−^CTR1^fl/fl^). Genotyping of the mice was performed via PCR amplification of genomic DNA obtained from tail tissue. The primers utilized for genotyping are provided in Table S2.

### 4.3 Construction of Renal‐Specific COMMD1 Inhibition Mice

Adeno‐associated viruses carrying shRNA targeting the COMMD1 gene (AAV9‐shCOMMD1) and the corresponding negative controls (AAV9‐shNC) were designed and purchased from Genomeditech Co., LTD, Shanghai, China). The mouse COMMD1 shRNA target sequence used was 5'‐ACAGCCCTGTTGCCATAATAG‐3'. The viruses were administered via intraparenchymal injections into the kidney, following a procedure previously described [35]. AAV‐mediated COMMD1 protein expression in the kidney was significantly reduced after one month, and the mice were then used in the following animal experiment.

### 4.4 Animals Experiment

Eight‐week‐old male C57BL/6J mice (23‐25 g) were housed under temperature‐controlled conditions with a 12‐h light/dark cycle and given standard chow. All procedures were approved by the Animal Ethics Committee of Tongji University School of Medicine (No. TJ‐HB‐LAC‐2023‐47).

Mice were subjected to unilateral renal ischemia/reperfusion injury without contralateral nephrectomy (IRI) surgery. Briefly, after anesthesia, the left kidney was exposed and the renal pedicle clamped with non‐traumatic microvascular clamps for 30 min to induce ischemia. Body temperature was maintained at 37°C throughout the procedure, and the contralateral kidney was left intact. Sham‐operated mice underwent identical procedures without renal pedicle clamping. Mice were euthanized 28 days post‐IRI surgery. Unilateral ureteral obstruction (UUO) was performed as described previously [36]. Following anesthesia, the left ureter was ligated with 5‐0 silk sutures. Sham‐operated controls underwent the same procedure without ligation. 14 days post‐surgery, mice were sacrificed, and kidney tissues were harvested for analysis.

For the TEC‐specific CTR1 knockout experiment, all mice were randomly assigned to four groups: sham‐operated WT mice (Cre^−^CTR1^fl/fl^+Sham), sham‐operated Cre^+^CTR1^fl/fl^ mice (Cre^+^CTR1^fl/fl^+Sham), IRI 28d‐operated WT mice (Cre^−^CTR1^fl/fl^+IRI 28d), and IRI 28d‐operated Cre^+^CTR1^fl/fl^ mice (Cre^+^CTR1^fl/fl^+IRI 28d). For AAV9‐shCOMMD1 intervention experiment, the mice were divided into four groups: sham‐operated mice treated with shNC (shNC+Sham), sham‐operated mice treated with shCOMMD1 (shCOMMD1+Sham), IRI mice treated with shNC (shNC+IRI 28d), and IRI mice treated with shCOMMD1 (shCOMMD1+IRI 28d). For the Tempol intervention experiment, mice subjected to IRI surgery received daily oral gavage of Tempol (HY‐100561, MCE, Shanghai, China) at 200 mg/kg for 28 days, while control mice received an equal volume of sterile water. All mice were euthanized 28 days after the respective interventions, and serum and kidney tissues were harvested for subsequent analyses.

### 4.5 Cell Culture and Treatment

Rat renal tubular epithelial cells (NRK‐52E) were purchased from FuHeng Biology (FH0417, Shanghai, China) and cultured in DMEM (Hyclone, Logan, Utah) supplemented with 5% fetal bovine serum at 37°C in a 5% CO2 incubator. Cells were serum‐starved for 12 h prior to treatment with 10 ng/mL TGF‐β1 (100‐21C‐10UG, PeproTech, USA) with or without Tempol or CuSO_4_ (C8027, Sigma‐Aldrich, USA) or TM (323446, Sigma‐Aldrich, USA) treatment. For specific assays, NRK‐52E cells were treated with CuSO_4_ for 48 h and treated with 10 µmol/L CHX (HY‐12320, MCE, Shanghai, China) for the indicated time points. For protein stability analysis, NRK‐52E cells were transfected with the pcDNA3.1‐COMMD1 plasmid (Genomeditech, Shanghai, China) for 48 h, followed by treatment with or without 20 µmol/L MG132 (HY‐13259, MCE, Shanghai, China) for 4 h prior to harvest.

Lentiviruses for CTR1 knockdown, COMMD1 knockdown, and COMMD1 overexpression were generated using respective plasmids purchased from Genomeditech (Shanghai, China). NRK‐52E cells were infected with lentiviruses: ZsGreen‐PURO‐shCTR1, ZsGreen‐PURO‐shCOMMD1, and ZsGreen‐PURO‐3×Flag‐COMMD1. The rat COMMD1 shRNA target sequence used was 5'‐GTCACGGCACTCAACTCAAAT‐3'. The rat CTR1 shRNA target sequence used was 5'‐GACCTACAATGGGTACCTA‐3'. Successfully transduced cells were selected with puromycin and subsequently harvested at designated time points following TGF‐β1 stimulation respectively.

### 4.6 Renal Histology and Immunohistochemistry

Mouse kidney tissues were fixed in 10% formalin, embedded in paraffin, and sliced into 4‐µm‐thick sections. hematoxylin‐eosin (HE) and Masson's trichrome staining were carried out following standard protocols. Interstitial fibrosis was assessed by evaluating areas of interstitial collagen deposition in Masson's trichrome staining as described previously [37].

To conduct immunohistochemical staining, tissue sections were deparaffinized in xylene, rehydrated in graded ethanol. Antigen retrieval was performed by microwaving the sections in Tris‐EDTA buffer (pH 9.0) for 20 min. Endogenous peroxidase activity was blocked with 3% hydrogen peroxide for 20 min, followed by blocking with 5% goat serum for 60 min at room temperature. Sections were incubated overnight at 4°C with an anti‐COMMD1 antibody (Proteintech, 67016‐1‐Ig), followed the next day by incubation with an HRP‐conjugated secondary antibody for 1 h at room temperature. Staining was visualized using a 3,3′‐diaminobenzidine (DAB) (SK4100, Vector) kit and observed and captured under a Nikon light microscope.

### 4.7 Immunofluorescence Staining

Routine processing steps for mouse kidney paraffin sections before immunofluorescence detection. Tissue sections were incubated with anti‐COMMD1 (67016‐1‐Ig, Proteintech) and rabbit anti‐AQP1 (20333‐1‐AP, Proteintech). Goat anti‐rabbit/mouse secondary antibody (goat anti‐mouse‐594, ab150120, Abcam; goat anti‐rabbit‐488, ab150081, Abcam) in blocking serum was added for an hour of incubation at room temperature. NRK‐52E cells were cultured in a confocal dish and fixed with 4% paraformaldehyde at room temperature for 20 min. Cells were then permeabilized with 0.3% Triton X‐100 for 20 min and blocked with 5% goat serum for 1 h. Subsequently, cells were incubated overnight at 4°C with anti‐COMMD1 (Proteintech, 67016‐1‐Ig), followed the next day by incubation with goat anti‐mouse secondary antibody (goat anti‐mouse‐594, ab150120, Abcam) at room temperature for 30 min shielded from light. Nuclei were stained with DAPI for 5 min. Fluorescence images were captured with a confocal microscope.

### 4.8 Western Blot

NRK‐52E cells and kidney tissues were harvested and lysed in lysis buffer for protein extraction. Protein concentrations were determined using the BCA assay, and equal amounts of protein were separated by SDS‐PAGE. The proteins were then transferred onto PVDF membrane (Millipore, Darmstadt, Germany) via electrophoresis. The membrane was then blocked with 5% nonfat milk for 1 h and incubated overnight at 4°C with the primary antibody. After incubation with secondary antibodies, protein bands were visualized using an enhanced chemiluminescence system (Amersham, UK). Band intensities were quantified using ImageJ and normalized to GAPDH. Primary antibodies applied were as follows: anti‐COMMD1 (67016‐1‐Ig, Proteintech), anti‐Collagen I (ab260043, abcam), anti‐FN (ab2413, abcam), anti‐p16^INK4A^ (sc‐1661, Santa Cruz), anti‐p21 (28248‐1‐AP, Proteintech), anti‐p21 (sc‐6246, Santa Cruz), anti‐SOD1 (67480‐1‐Ig, Proteintech), anti‐SOD1 (ab13498, abcam), anti‐CTR1 (ab129067, abcam), anti‐GAPDH (bsm‐33033 M, Bioss).

### 4.9 RNA Extraction and Quantitative Real‐Time PCR

Total RNA was isolated from cells and kidney tissues using Trizol reagent (Thermo Scientific, Wilmington, MA, USA) following the manufacturer's protocol. The extracted RNA was reverse transcribed into cDNA using the RT reagent kit (Takara, Japan). Real‐time PCR was subsequently performed with SYBR Green Master Mix (Takara, Japan) on the ABI Prism 7500 Sequence Detection System (Foster City, CA, USA). Relative expression levels were calculated using the 2^−ΔΔCt^ method and normalized to GAPDH expression. The sequence‐specific primers used for PCR amplification are listed in Table S3.

### 4.10 Copper Content Measurement

Samples from cells and kidney tissues were collected and analyzed using Inductively Coupled Plasma Mass Spectrometry (ICP‐MS). A range of standard copper solutions with different concentrations was prepared and diluted in a 2% nitric acid solution. The samples were dissolved in concentrated nitric acid and heated in a metal bath at 100°C until all suspended particles completely dissolved, yielding a clear solution. Afterward, the samples were diluted with purified water to reduce the concentration of the nitric acid to about 2%. Copper concentration was determined by ICP‐MS using a NexION 5000G instrument (PerkinElmer, USA) and calculated based on the standard curve of copper levels [38, 39].

### 4.11 Coppersensor‐1(CS1) Staining

Coppersensor‐1 (CS1) is a membrane‐permeable fluorescent dye with picomolar affinity for Cu^+^ and high selectivity over other cellular metal ions. It allows for the sensitive and selective detection of Cu^+^ in biological samples, including live cells. NRK‐52E cells following the specified treatment were washed with PBS, fixed in 4% paraformaldehyde, and permeabilized with 0.1% Triton‐X 100, with each step followed by three PBS washes. The cells were then incubated with 5 µM CS1 at 37°C in the dark for 20 min, washed with PBS, mounted with DAPI anti‐fade medium, and observed under a fluorescence microscope.

### 4.12 SOD1 Activity Detection

The activity of SOD1 in NRK‐52E cells and kidney tissues was assessed using commercial assay kits (Beyotime Institute of Biotechnology, China) in accordance with the manufacturer's instructions. Sample absorbance was measured at 450 nm using a microplate reader, and the data were reported as U/mg protein.

### 4.13 Copper Incubation, Desalting, and Assessment of SOD1 Cu/Zn Occupancy

To evaluate the effect of excess copper on the metal occupancy, the recombinant human SOD1 (ACROBiosystems, SO1‐H5148) was incubated with CuSO_4_ at a two‐fold molar excess relative to the theoretical copper content of dimeric SOD1 for 30 min at room temperature. Control samples received an equivalent volume of metal‐free ultrapure water. Two blanks (storage buffer alone and CuSO_4_‐containing buffer without protein) were included to assess background metals and removal efficiency. Unbound metals were removed using Zeba Spin Desalting Columns (Thermo Fisher Scientific, 89883) pre‐equilibrated with metal‐free buffer, and samples were centrifuged at 1,500 × g for 2 min to collect desalted protein fractions. Protein concentrations were subsequently determined by BCA assay. Desalted samples were digested with trace metal‐grade nitric acid, diluted with metal‐free ultrapure water to a final volume of 5 mL containing 2% nitric acid. Copper and zinc concentrations were analyzed by ICP‐MS, and Cu/Zn ratios were calculated accordingly. Blank controls showed no detectable Cu or Zn signals following the desalting procedure.

### 4.14 Visualization of SOD1 Monomers and Dimers

SOD1 monomers and dimers were visualized using a protocol previously described [29, 40]. In brief, cells were washed with PBS and lysed in a buffer containing protease inhibitors. Protein samples were incubated at 37°C for 30 min in a sample buffer without reductant and analyzed using SDS‐PAGE followed by immunoblotting.

### 4.15 Dihydroethidium (DHE) Staining

To evaluate ROS levels in cells and kidney tissues, dihydroethidium (DHE) staining was performed using the Reactive Oxygen Species Assay Kit for Superoxide Anion (Beyotime Institute of Biotechnology, China). DHE powder was dissolved in dimethyl sulfoxide, and cryosections or NRK‐52E cells were incubated with the DHE solution at 37°C for 30 min. Fluorescence was observed using a Nikon microscope imaging system. Fluorescence intensity was quantified using Image‐Pro Plus software.

### 4.16 SA‐β‐Gal Staining

Following the manufacturer's instructions, cultured cells and 5 µm frozen kidney sections were fixed and stained overnight at 37°C without CO2 using an SA‐β‐gal staining kit (Beyotime Institute of Biotechnology, China). SA‐β‐gal activity was quantified using Image‐Pro Plus software.

### 4.17 Data Independent Acquisition (DIA) Proteomics

NRK‐52E cells were treated with or without 40 µM CuSO_4_ for 48 h (n = 3 per group), followed by cell harvest, protein extraction, and digestion using the filter‐aided sample preparation (FASP) method. Peptides from each sample were separated using a Vanquish Neo UHPLC system coupled with an Orbitrap Astral mass spectrometer (Thermo Scientific). Peptides were separated on a µPAC Neo High Throughput analytical column using a gradient of up to 45% acetonitrile. DIA raw data were processed using DIA‐NN software for protein identification and quantification. The UniProt reference proteome for Rattus norvegicus (taxonomy ID: 10116), downloaded on 31 March 2026, was used for spectral library generation and protein identification. SOD1‐interacting proteins were curated from the STRING and BioGRID databases and mapped onto the DIA proteomics dataset for expression analysis. Within this subset, the entry with the highest peptide support was retained for genes with multiple entries. Statistical significance was defined as |log2 fold change (log2FC)| ≥ 1 and an adjusted *p*‐value (false discovery rate, FDR) < 0.05, calculated using the Benjamini‐Hochberg method.

### 4.18 Co‐Immunoprecipitation (Co‐IP)

NRK‐52E cells were subjected to different treatments before harvesting, depending on the study's objectives. The cells were then lysed using Western blot and IP lysis buffer (Beyotime Institute of Biotechnology, China) supplemented with a protease inhibitor cocktail (Solarbio, China). The lysate was centrifuged at 12 000 rpm for 10 min at 4°C to collect the supernatant. The supernatant was incubated overnight at 4°C with anti‐COMMD1 (11938‐1‐AP, Proteintech) or anti‐IgG (Proteintech) under gentle vertical rotation. Subsequently, the complex was incubated overnight at 4°C with Protein A/G PLUS‐Agarose (sc‐2003, Santa Cruz, Dallas, Texas). Afterward, the precipitates were washed and eluted for Western blot analysis.

### 4.19 Protein Structure

The protein structures of SOD1 and COMMD1 were predicted using AlphaFold3 modeling based on their amino acid sequences retrieved from the UniProt database [41]. The UniProt accession numbers for the referenced sequences are as follows: SOD1 (P07632) and COMMD1 (B0BNB1). The crystal structures of the SOD1‐SOD1 and COMMD1‐SOD1 complexes, as predicted by AlphaFold3 modeling, were utilized for molecular dynamics (MD) simulations. Additionally, an additional copper ion was added to SOD1‐SOD1 system and COMMD1‐SOD1 system to develop two new systems for MD simulations.

### 4.20 Molecular Dynamics Simulations

This study involves four molecular dynamics simulation systems: (1) SOD1‐SOD1‐normal copper, simulating SOD1 subunit interacting with itself under normal copper conditions; (2) COMMD1‐SOD1‐normal copper, simulating COMMD1 interacting with SOD1 subunit under normal copper conditions; (3) SOD1‐SOD1‐copper overload, simulating SOD1‐SOD1 under copper overload conditions (namely with an added copper ion); and (4) COMMD1‐SOD1‐copper overload, simulating COMMD1‐SOD1 under copper overload conditions (namely with an added copper ion).

MD simulations were conducted using Gromacs 2021.7 under isothermal‐isobaric conditions with periodic boundaries [42, 43]. The Amber99SB force field and SPC water model were applied [44]. Hydrogen bonds were constrained with the LINCS algorithm using a 2 fs time step. Electrostatic interactions were computed using the PME method with a 14 Å cutoff, updated every 50 steps. Temperature (298.15 K) and pressure (1 bar) were controlled using the V‐rescale thermostat and Parrinello‐Rahman barostat, respectively. Energy minimization was performed using the steepest descent method, followed by 100 ps of NVT and NPT equilibration at 298.15 K. A 100 ns production simulation was then performed, saving configurations every 50 ps. Results were visualized using Gromacs tools and VMD.

We analyzed the molecular dynamics trajectories of four molecular dynamics simulation systems. The analysis focused on RMSD, RMSF, protein secondary structure, protein‐ligand interactions, and structural comparisons at six time points (0, 20, 40, 60, 80, and 100 ns). Additionally, we calculated the binding free energy between SOD1 and COMMD1 using the MM/GBSA method.

### 4.21 Statistical Analysis

Statistical analyses were performed using GraphPad Prism software (version 10). Data normality was assessed using the Shapiro‐Wilk test. For comparisons between two groups, a two‐tailed unpaired Student's t‐test was used for normally distributed data, whereas the Mann‐Whitney U test was applied for non‐normally distributed data. Comparisons among multiple groups were performed using one‐way analysis of variance (ANOVA) followed by Tukey's post hoc test. Correlation analysis was performed using Pearson's correlation test. Data are presented as mean ± SEM, and the sample size (n) for each experiment is indicated in the corresponding figure legends. A *p* value < 0.05 was considered statistically significant.

## Author Contributions

Y.Q.L, Y.Y.Z, and C.Y conducted the conceptualization and design of the study. Y.Q.L, J.L, W.Q.Z, and Y.Y.N contributed to data acquisition, formal analysis and methodology. Y.Q.L performed investigation. Y.Z and Y.G.L performed formal analysis. Y.Y.Z and C.Y revised the manuscript. C.Y supervised the project and provided financial, technical, and material support.

## Conflicts of Interest

The authors declare no conflicts of interest.

## Supporting information




**Supporting File 1**: advs76391‐sup‐0001‐SuppMat.docx.

**Supporting File 2**: advs76391‐sup‐0002‐FigureS1.tif.

**Supporting File 3**: advs76391‐sup‐0003‐FigureS2.tif.

**Supporting File 4**: advs76391‐sup‐0004‐FigureS3.tif.

**Supporting File 5**: advs76391‐sup‐0005‐FigureS4.tif.

**Supporting File 6**: advs76391‐sup‐0006‐FigureS5.tif.

**Supporting File 7**: advs76391‐sup‐0007‐FigureS6.tif.

**Supporting File 8**: advs76391‐sup‐0008‐FigureS7.tif.

**Supporting File 9**: advs76391‐sup‐0009‐FigureS8.tif.

**Supporting File 10**: advs76391‐sup‐0010‐FigureS9.tif.

**Supporting File 11**: advs76391‐sup‐0011‐FigureS10.tif.

**Supporting File 12**: advs76391‐sup‐0012‐FigureS11.tif.

## Data Availability

The data that support the findings of this study are available from the corresponding author upon reasonable request.
