# Supplementary material for: Pathological Copper Overload Reprograms SOD1 Activation via COMMD1 to Promote Senescence and Fibrosis
Source: Adv Sci (Weinh). 2026 Jul 2;13(54):e76391. doi: 10.1002/advs.76391 (PMC13334582; doi:10.1002/advs.76391)
Supplement: Supplementary file 1 [file ADVS-13-e76391-s002.docx]

**Supporting Information**

**Pathological Copper Overload Reprograms SOD1 Activation via COMMD1 to Promote Senescence and Fibrosis**

Yuqing Liu, Jing Liu, Wenqian Zhou, Yangyang Niu, Yan Zheng, Yiguo Liu, Yingying Zhang*, Chen Yu*

Department of Nephrology, Tongji Hospital, School of Medicine, Tongji University, Shanghai, 200092, China

*Corresponding author: Yingying Zhang ([idklaa@hotmial.com](mailto:idklaa@hotmial.com)) and Chen Yu ([yuchen@tongji.edu.cn](mailto:yuchen@tongji.edu.cn))

*These authors contributed equally as corresponding authors.

**This file includes:**

Figs. S1 to S11

Tables S1 to S3

**Figure S1. Tempol, a superoxide dismutase mimetic, reduces ROS levels and alleviates renal senescence and fibrosis.** **(A)** Experimental schematic of Tempol administration in IRI-induced renal fibrosis. **(B)** Representative DHE staining images and quantification in the kidneys of Sham and IRI mice with or without Tempol treatment (n = 6). Bar = 100 μm. **(C)** Representative micrographs of SA-β-gal, HE and Masson's trichrome staining in the kidneys from different groups of mice. Quantification of SA-β-gal positive areas based on SA-β-gal staining and fibrotic areas based on Masson staining (n = 6). Bar = 50 μm. **(D)** Western blot images and quantitative analysis of p16^INK4A^, p21, COL1A1 and FN in the kidneys from different groups of mice (n = 6). **(E)** Representative DHE images and quantification of the average DHE fluorescence intensity, normalized to the CTL group, in NRK-52E cells (n = 3). Bar = 50 μm. **(F)** Representative images and quantification of SA-β-gal staining in different groups of NRK-52E cells (n = 3). Bar = 50 μm. **(G)** Western blot images and quantitative analysis of p16^INK4A^, p21, COL1A1 and FN across different groups (n = 3). Data were shown as the mean ± SEM. ***P < 0.001 versus Sham group or versus CTL group. ^###^P < 0.001 versus IRI 28d or versus TGF-β1 group. Statistical analysis was performed using one-way ANOVA followed by Tukey's post hoc test.

**Figure S2. Intracellular copper overload, cellular senescence and renal fibrosis in the kidney tissues of UUO mice.** **(A)** Copper content was detected by ICP-MS in the kidney tissues of mice with or without UUO surgery (n = 6). **(B)** Representative images and quantification of CS1-stained kidney sections (n = 6). Bar = 10 μm. **(C)** Representative SA-β-gal, HE, Masson's trichrome staining micrographs of kidneys from mice with or without UUO surgery. Quantification of SA-β-gal positive areas based on SA-β-gal staining (n = 6). Bar = 50 μm. **(D)** Western blot images and quantification of p16^INK4A^, p21, COL1A1 and FN across different groups (n = 6). Data were shown as the mean ± SEM. ***P < 0.001 versus Sham group. Statistical analysis was performed using two-tailed unpaired Student’s t-test.

**Figure S3. Generation and identification of TEC-specific CTR1 knockout mice and validation of CTR1 knockdown efficiency in NRK-52E cells. (A)** Schematic of the generation of renal TEC-specific CTR1 knockout mice. **(B)** Representative images of different genotypes assessed by PCR amplification of DNA isolated from mice tail tissue. **(C)** Western blot images and quantification of CTR1 in the kidneys of Cre^-^CTR1^fl/fl^ and Cre^+^CTR1^fl/fl^ mice (n = 6). **(D)** Assessment of CTR1 knockout efficiency by immunohistochemistry in renal sections of Cre^-^CTR1^fl/fl^ and Cre^+^CTR1^fl/fl^ mice (n = 6). **(E)** Validation of CTR1 knockdown efficiency by western blot with quantitative results in NRK-52E cells transfected with control shRNA (shNC) and shCTR1 (n = 3). **(F)** Quantification of CS1 staining in NRK-52E cells across different groups (n = 3). **(G)** Quantification of the average DHE fluorescence intensity, normalized to shNC group, in different groups of NRK-52E cells (n = 3). **(H)** Quantification of SA-β-gal positive cells based on SA-β-gal staining in NRK-52E cells across different groups (n = 3). Data were shown as the mean ± SEM. ***P < 0.001 versus Cre^-^CTR1^fl/fl^ group or versus shNC group. ###P < 0.001 versus shNC+TGF-β1 group. Statistical analysis was performed using two-tailed unpaired Student’s t-test or one-way ANOVA followed by Tukey's post hoc test.

**Figure S4.** **Quality control and global proteomic alterations revealed by DIA-based quantitative proteomics. (A)** Principal component analysis (PCA) of all quantified proteins obtained from DIA mass spectrometry between control and CuSO_4_-treated groups (n = 3). **(B)** Volcano plot of differentially expressed proteins between control and CuSO₄-treated groups (n = 3). Differentially expressed proteins were defined by thresholds of |log2FC| ≥ 1 and FDR< 0.05. **(C)** Heatmap of differentially expressed proteins based on Z-score normalized intensity values (n = 3). Each row represents a protein and each column represents a biological replicate.

**Figure S5. SOD1-centered protein-protein interaction (PPI) network from STRING and BioGRID databases.** The SOD1-centered PPI network was constructed using interaction data from STRING and BioGRID databases. For STRING, interactions with a confidence score > 0.7 were retained. For BioGRID, interactions with low-throughput experiments and with at least two independent pieces of evidence were included. Nodes represent proteins, and edges indicate protein-protein interactions.

**Figure S6. COMMD1 mRNA levels and protein stability in CuSO_4_-treated NRK-52E cells.** **(A)** The mRNA levels of COMMD1 in NRK52E cells treated with different doses of TGF-β1 (n = 3). **(B)** The mRNA levels of COMMD1 in TGF-β1-treated NRK52E cells with or without copper chelator tetrathiomolybdate (TM) (n = 3). **(C)** NRK52E cells pretreated with CuSO₄ for 48 h were subjected to cycloheximide (CHX) and harvested at 0, 2, 4, and 8 h (n = 3). Data were shown as the mean ± SEM. **P < 0.01, ***P < 0.001 versus CTL group. ^###^P < 0.001 versus TGF-β1 group. Statistical analysis was performed using two-tailed unpaired Student’s t-test or one-way ANOVA followed by Tukey's post hoc test.

**Figure S7.** **COMMD1 is upregulated in fibrotic kidneys of UUO mice and TGF-β1–treated NRK-52E cells. (A)** Representative immunohistochemical staining of COMMD1 and HE, Masson's trichrome staining micrographs in kidney sections of mice with or without UUO surgery (n = 6). Bar = 50 μm. **(B, C)** Western blot images and quantitative results of COMMD1, COL1A1 and FN in the kidneys of mice with or without UUO surgery (n = 6). **(D)** Representative immunofluorescence staining micrographs of COMMD1 in NRK52E cells treated with or without TGF-β1 (n = 3). Bar = 20 μm. Data were shown as the mean ± SEM. ***P < 0.001 versus Sham group. Statistical analysis was performed using two-tailed unpaired Student’s t-test.

**Figure S8. Assessment of COMMD1 knockdown and overexpression efficiency in NRK-52E cells. (A)** Validation of COMMD1 knockdown by western blot with quantitative analysis in NRK-52E cells transfected with control shRNA (shNC), shCOMMD1-1 (sh-1), shCOMMD1-2 (sh-2) and shCOMMD1-3 (sh-3), n = 3. **(B)** COMMD1 overexpression in NRK-52E cells was confirmed by Western blot with quantitative analysis. (n = 3). Data were shown as the mean ± SEM. ***P < 0.001 versus shNC group or Vector group. Statistical analysis was performed using two-tailed unpaired Student’s t-test or one-way ANOVA followed by Tukey's post hoc test.

**Figure S9.** **COMMD1 overexpression does not alter total SOD1 protein abundance in the presence or absence of MG132.** Western blot images of SOD1 protein expression with quantitative results in both vector or COMMD1 overexpression plasmids transfected NRK-52E cells, followed by 4-hour proteasome inhibitor (MG132) treatment prior to harvest (n = 3). Data were shown as the mean ± SEM. ns versus COMMD1 OE group. Statistical analysis was performed using one-way ANOVA followed by Tukey's post hoc test.

**Figure S10. Analysis of molecular dynamics simulation results. (A)** Root Mean Square Deviation (RMSD) was measured under four molecular dynamics simulation systems to evaluate system stability. **(B)** Representative 3D conformations of the SOD1-SOD1 complex under different molecular dynamics simulation systems were evaluated from the same perspective at six time points (0, 20, 40, 60, 80, and 100 ns). Blue and green indicate the two identical SOD1 subunits respectively. **(C)** Secondary structure analysis of the COMMD1-SOD1 and SOD1-SOD1 complexes across different simulation systems. **(D)** The RMSF of all amino acid residues in SOD1-SOD1 complex was assessed under different simulation systems.

**Figure S11.** **Validation of COMMD1 knockdown efficiency in kidney tissues. (A)** Assessment of COMMD1 knockdown efficiency by RT-PCR in kidney tissues transfected with AAV9-shNC or AAV9-shCOMMD1 (n = 6). **(B)** Evaluation of COMMD1 knockdown efficiency by western blot with quantitative results in kidney tissues transfected with AAV9-shNC or AAV9-shCOMMD1 (n = 6). Data were shown as the mean ± SEM. ***P < 0.001 versus shNC group. Statistical analysis was performed using two-tailed unpaired Student’s t-test.

**Table S1. Quantitative analysis of the SOD1-centered interaction landscape by DIA proteomics.**

| **Gene** | **Accession** | **Mean (CTL)** | **Mean (CuSO_4_)** | **Log2FC** | **P value** | **FDR** |
| --- | --- | --- | --- | --- | --- | --- |
| Commd1 | A0ABK0LU39 | 68535.45 | 240192.32 | 1.8093 | 0.0021 | 0.0483 |
| Hspa5 | P06761 | 16730204.11 | 22642221.27 | 0.4366 | 0.0012 | 0.0391 |
| Derl1 | A0A8I6AIT0 | 1207790.80 | 1433347.36 | 0.2470 | 0.0043 | 0.0670 |
| Ccs | Q9JK72 | 234854.08 | 260765.81 | 0.1510 | 0.0056 | 0.0746 |
| Hspa8 | P63018 | 17911615.54 | 19404615.30 | 0.1155 | 0.0096 | 0.0959 |
| Hspa4 | O88600 | 1701511.83 | 1833775.26 | 0.1080 | 0.0130 | 0.1112 |
| Bcl2 | P49950 | 100812.36 | 63706.56 | -0.6622 | 0.0609 | 0.2516 |
| Sod2 | P07895 | 1293298.06 | 1068205.30 | -0.2759 | 0.0181 | 0.1323 |
| Park7 | O88767 | 2246602.74 | 2105617.97 | -0.0935 | 0.1356 | 0.3833 |
| Optn | Q8R5M4 | 11185.21 | 10506.38 | -0.0903 | 0.5968 | 0.7918 |
| Vdac1 | Q9Z2L0 | 9794033.70 | 9226024.89 | -0.0862 | 0.2243 | 0.4983 |
| Tardbp | A0A8I6GLU8 | 3503078.11 | 3334502.26 | -0.0712 | 0.0599 | 0.2500 |

**Table S2. The primer sequence used for genotyping.**

| **Gene** | **Forward primer** | **Reverse primer** |
| --- | --- | --- |
| CTR1 flox | CTCACACATGCTAAGCAAATGATGTC | CTACATTCAAACACACCAGGGAAGA |
| Ggt1-Cre | CATCACATCAGGCACCCCAGAA | GAACATCTTCAGGTTCTGCGGGA |

**Table S3. Primer sequences used for RT-PCR.**

| **Gene** | **Forward primer** | **Reverse primer** |
| --- | --- | --- |
| Mouse-SOD1 | AACCAGTTGTGTTGTCAGGAC | CCACCATGTTTCTTAGAGTGAGG |
| Mouse-COMMD1 | TCAACCAGTTAGAGGCATTCCT | GAGTGCCGTGACTGAGACTTG |
| Mouse-GAPDH | AGGTCGGTGTGAACGGATTTG | TGTAGACCATGTAGTTGAGGTCA |
| Rat-SOD1 | GGATGAAGAGAGGCATGTTGGAGAC | GACCACCATAGTACGGCCAATGATG |
| Rat-COMMD1 | CAAGTCCCAGTCACGGCACTC | CGCTGATACTCTCTTCCACCTCTG |
| Rat-GAPDH | CGGCAACTTCAACGGCACAGTCA | GGTTTCTCCAGGCGGCATGTCA |
