# Supplementary figures and images for: Pathological Copper Overload Reprograms SOD1 Activation via COMMD1 to Promote Senescence and Fibrosis
Source: Adv Sci (Weinh). 2026 Jul 2:e76391. Online ahead of print. doi: 10.1002/advs.76391 (PMC13334582; doi:10.1002/advs.76391)

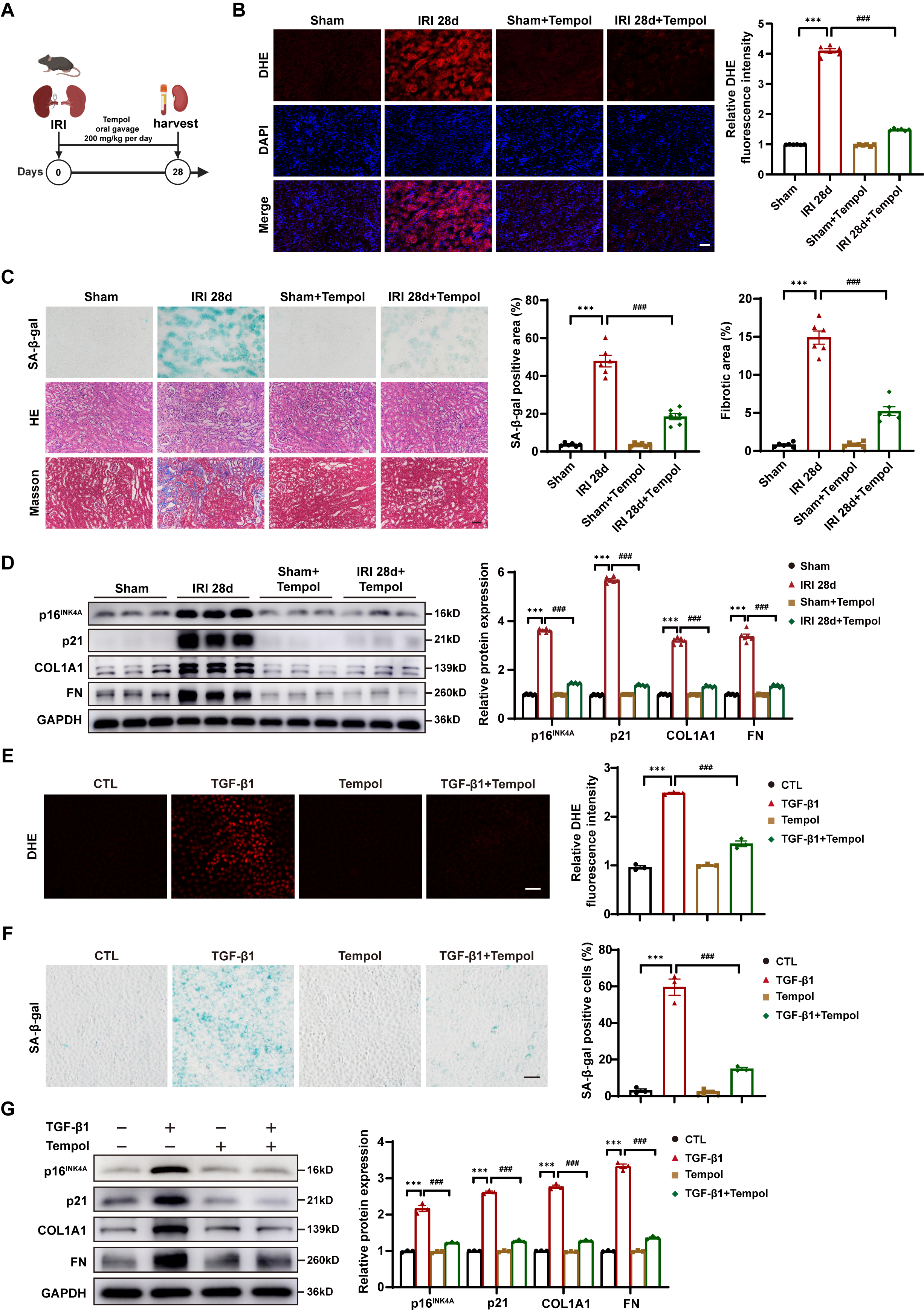

Supplement: Supplementary file 2 — Supporting File 2: advs76391‐sup‐0002‐FigureS1.tif. [file ADVS-9999-e76391-s012.tif]

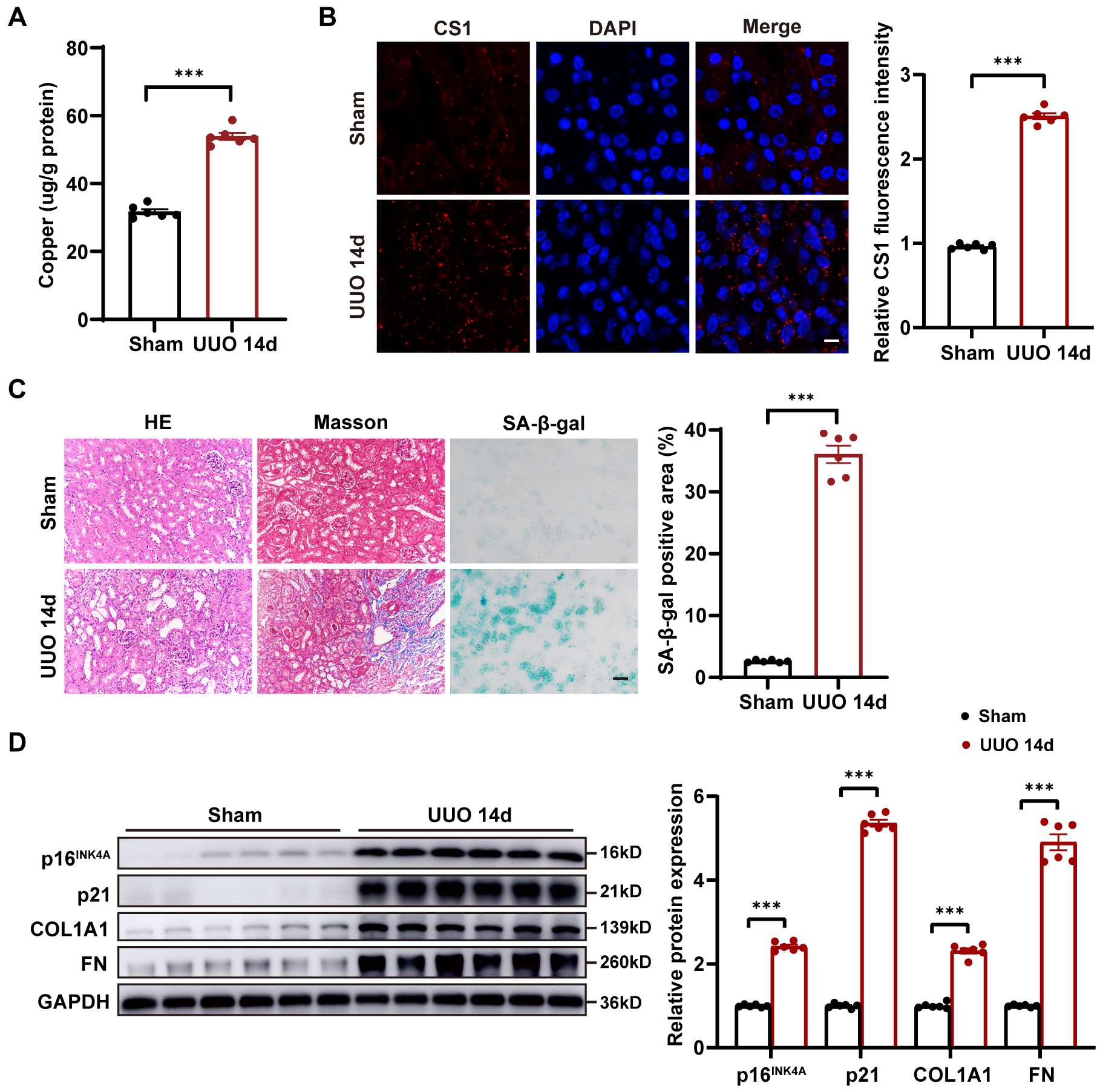

Supplement: Supplementary file 3 — Supporting File 3: advs76391‐sup‐0003‐FigureS2.tif. [file ADVS-9999-e76391-s001.tif]

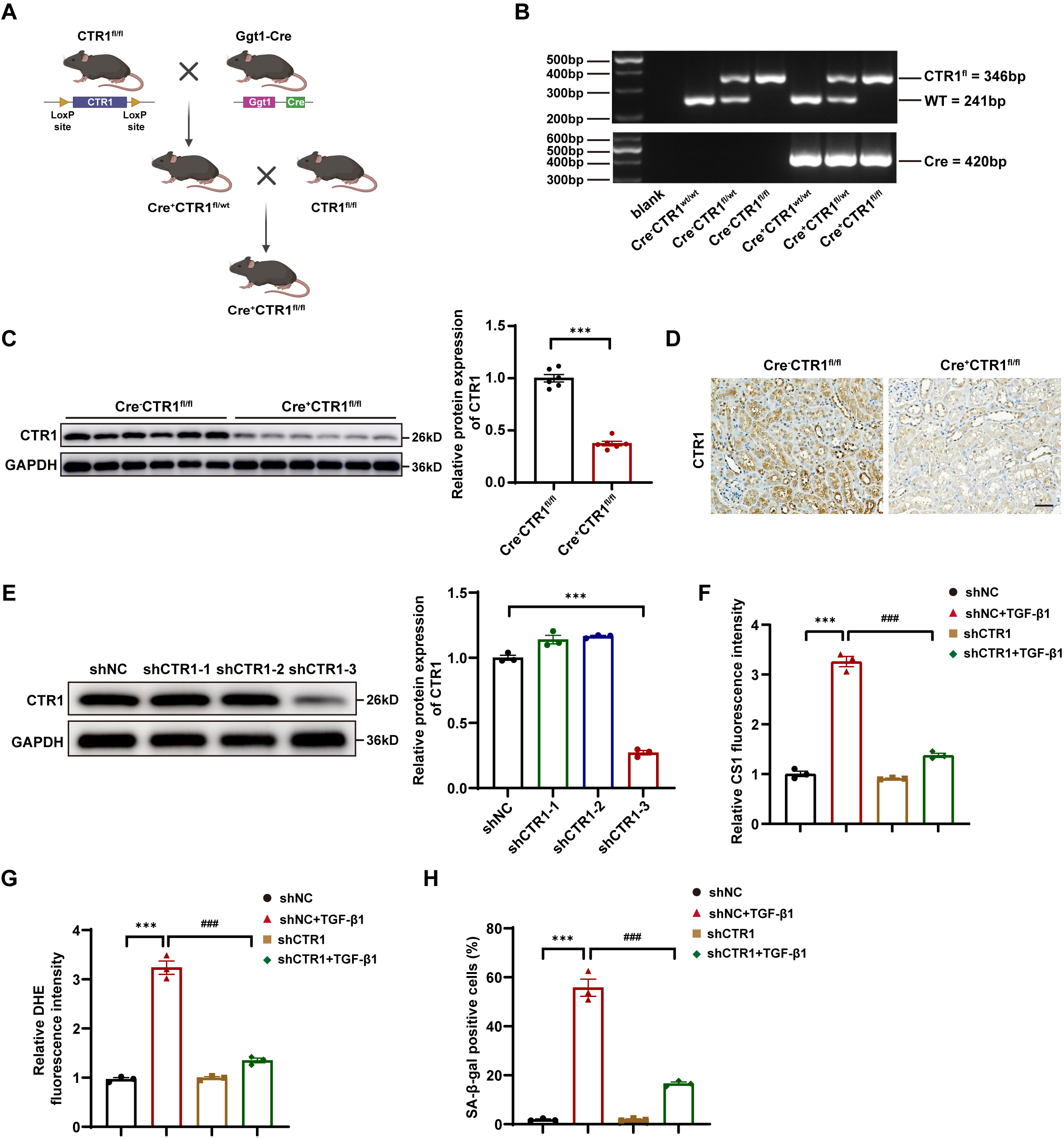

Supplement: Supplementary file 4 — Supporting File 4: advs76391‐sup‐0004‐FigureS3.tif. [file ADVS-9999-e76391-s004.tif]

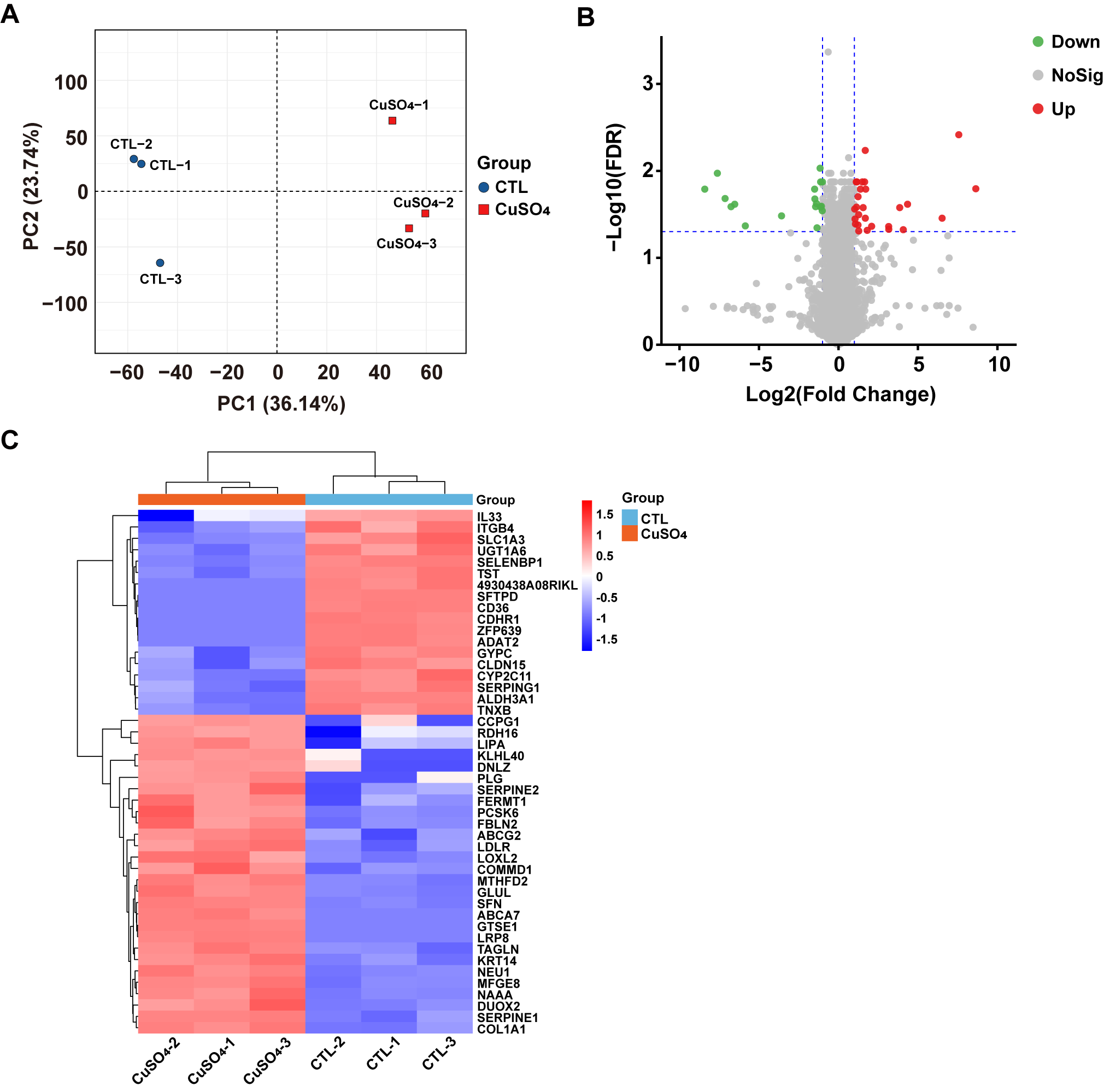

Supplement: Supplementary file 5 — Supporting File 5: advs76391‐sup‐0005‐FigureS4.tif. [file ADVS-9999-e76391-s005.tif]

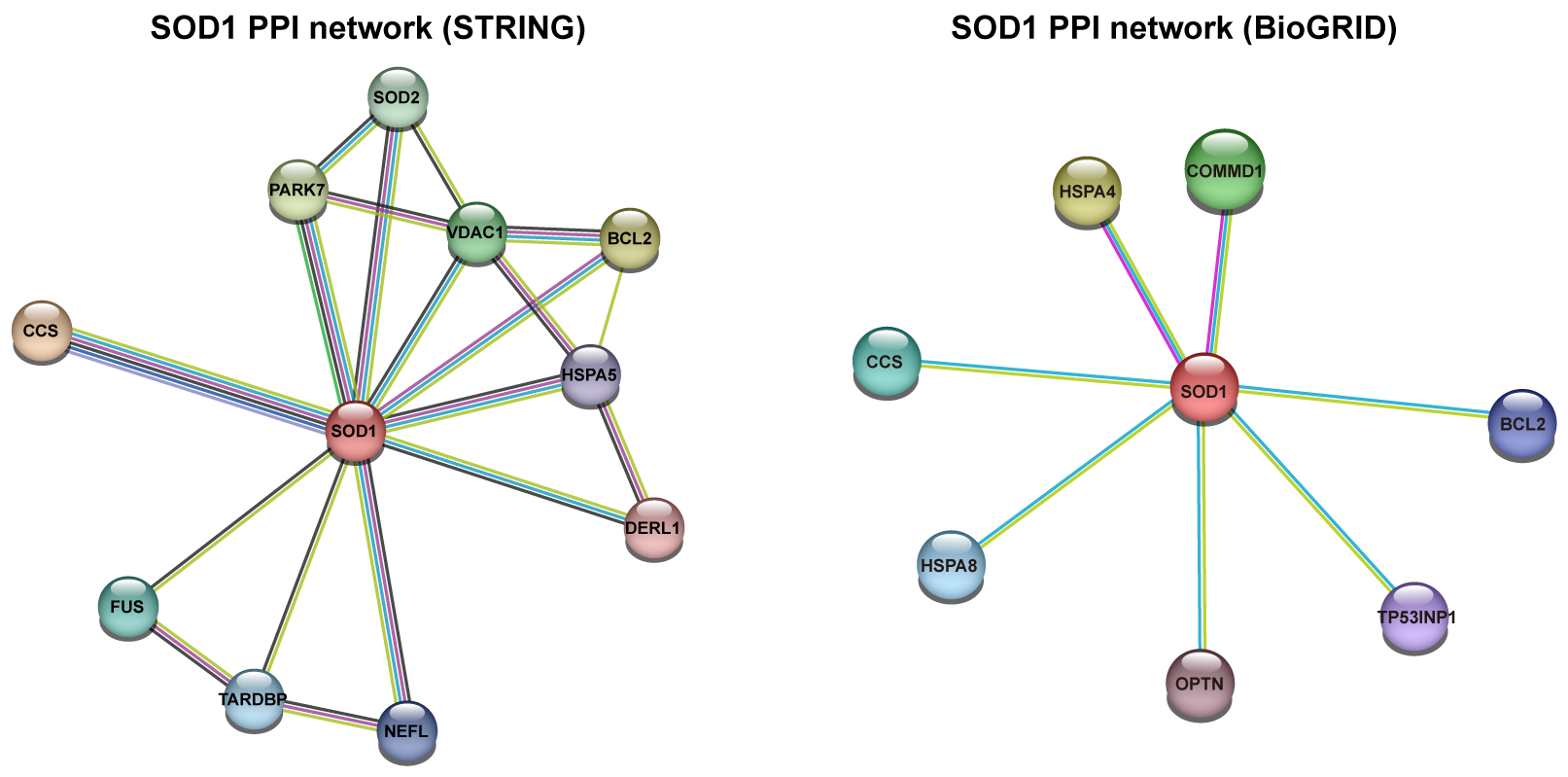

Supplement: Supplementary file 6 — Supporting File 6: advs76391‐sup‐0006‐FigureS5.tif. [file ADVS-9999-e76391-s003.tif]

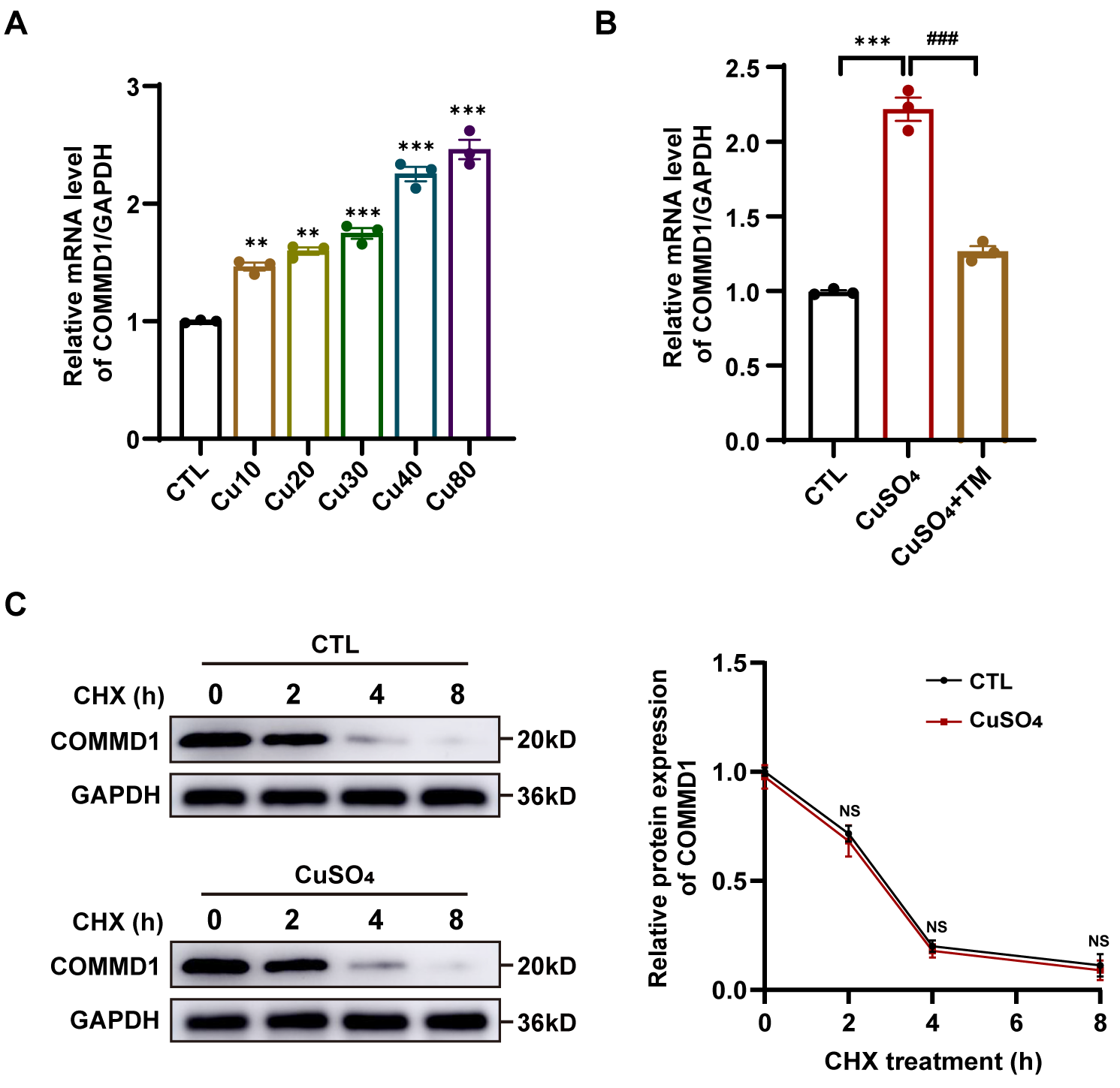

Supplement: Supplementary file 7 — Supporting File 7: advs76391‐sup‐0007‐FigureS6.tif. [file ADVS-9999-e76391-s006.tif]

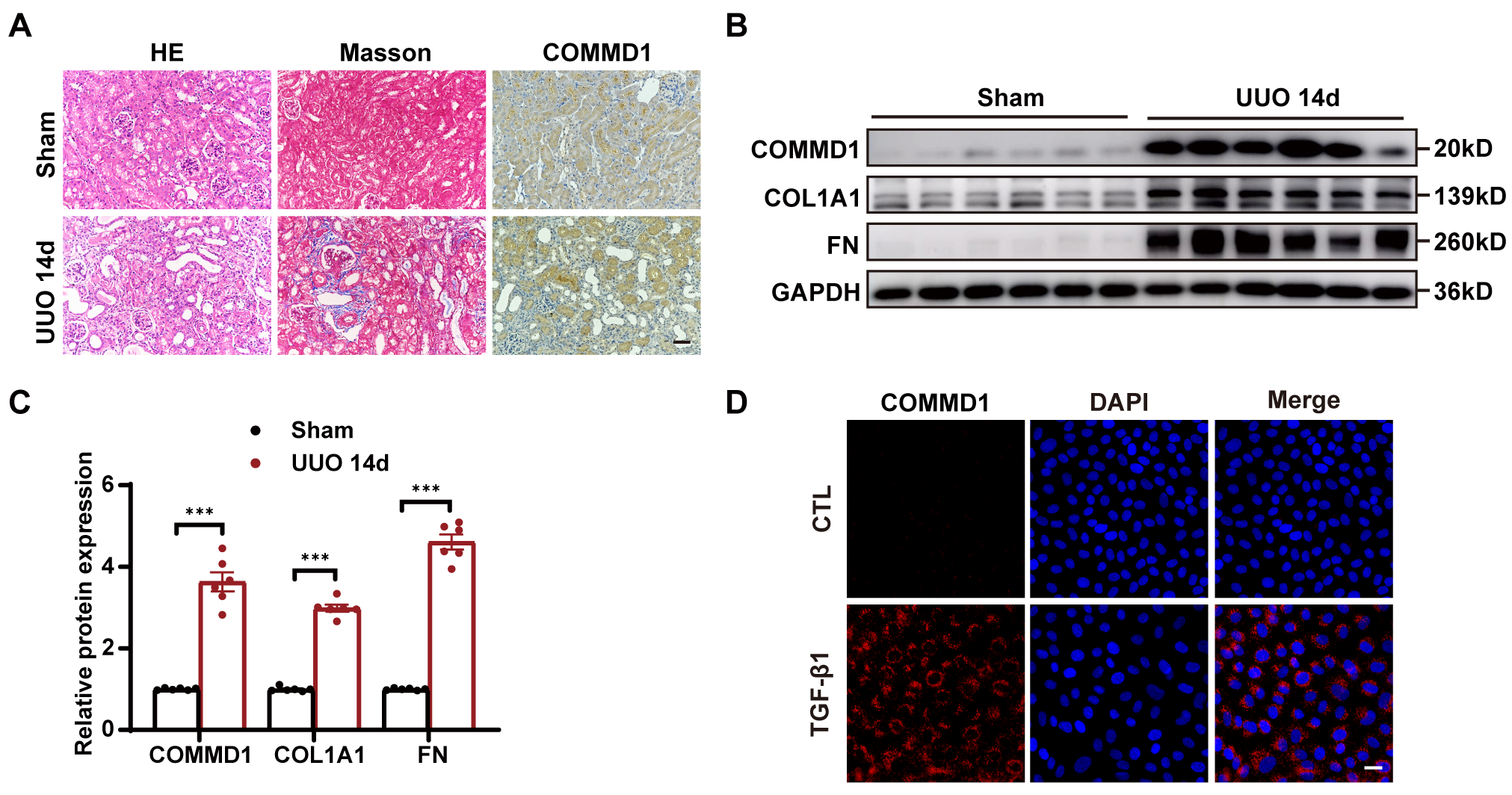

Supplement: Supplementary file 8 — Supporting File 8: advs76391‐sup‐0008‐FigureS7.tif. [file ADVS-9999-e76391-s010.tif]

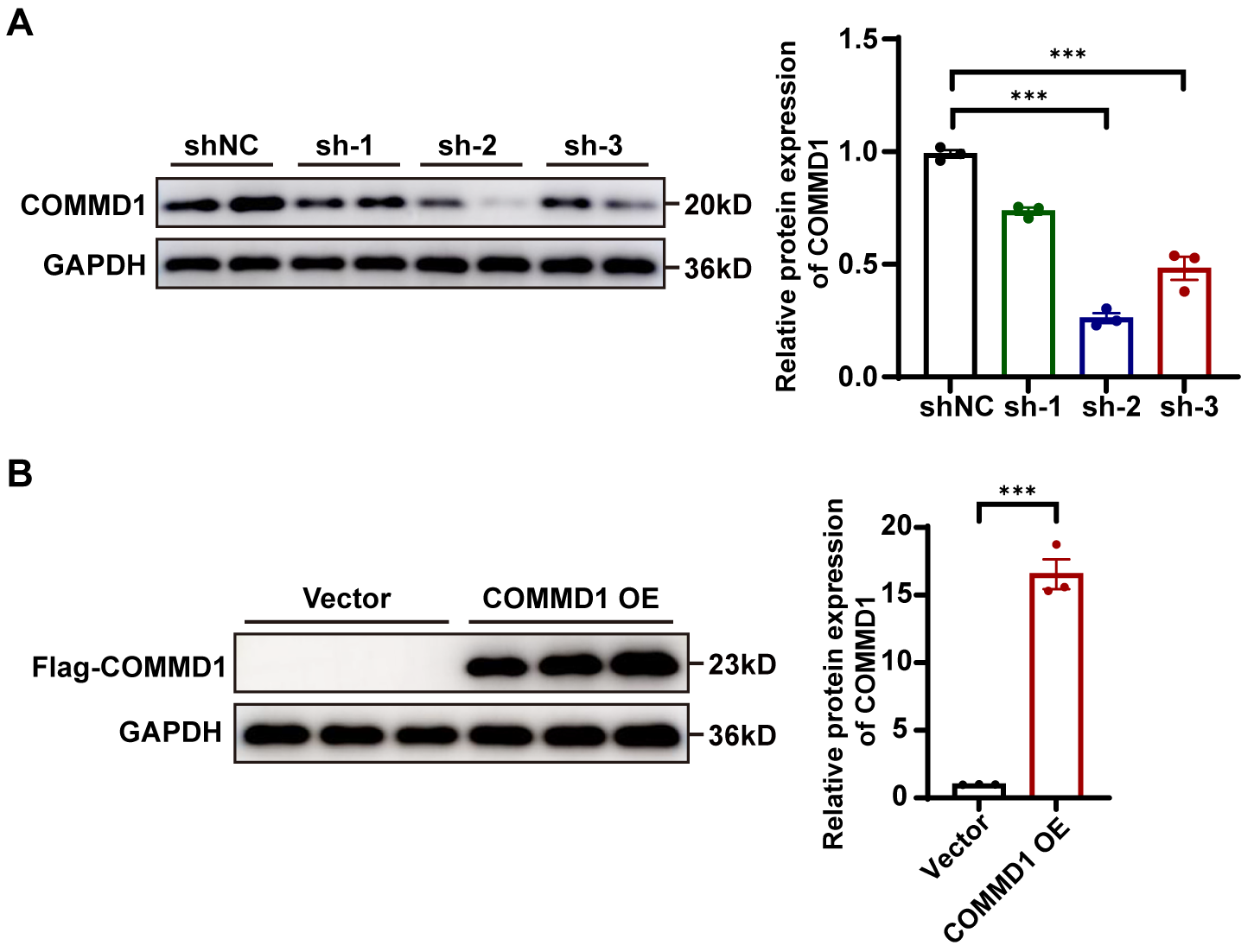

Supplement: Supplementary file 9 — Supporting File 9: advs76391‐sup‐0009‐FigureS8.tif. [file ADVS-9999-e76391-s009.tif]

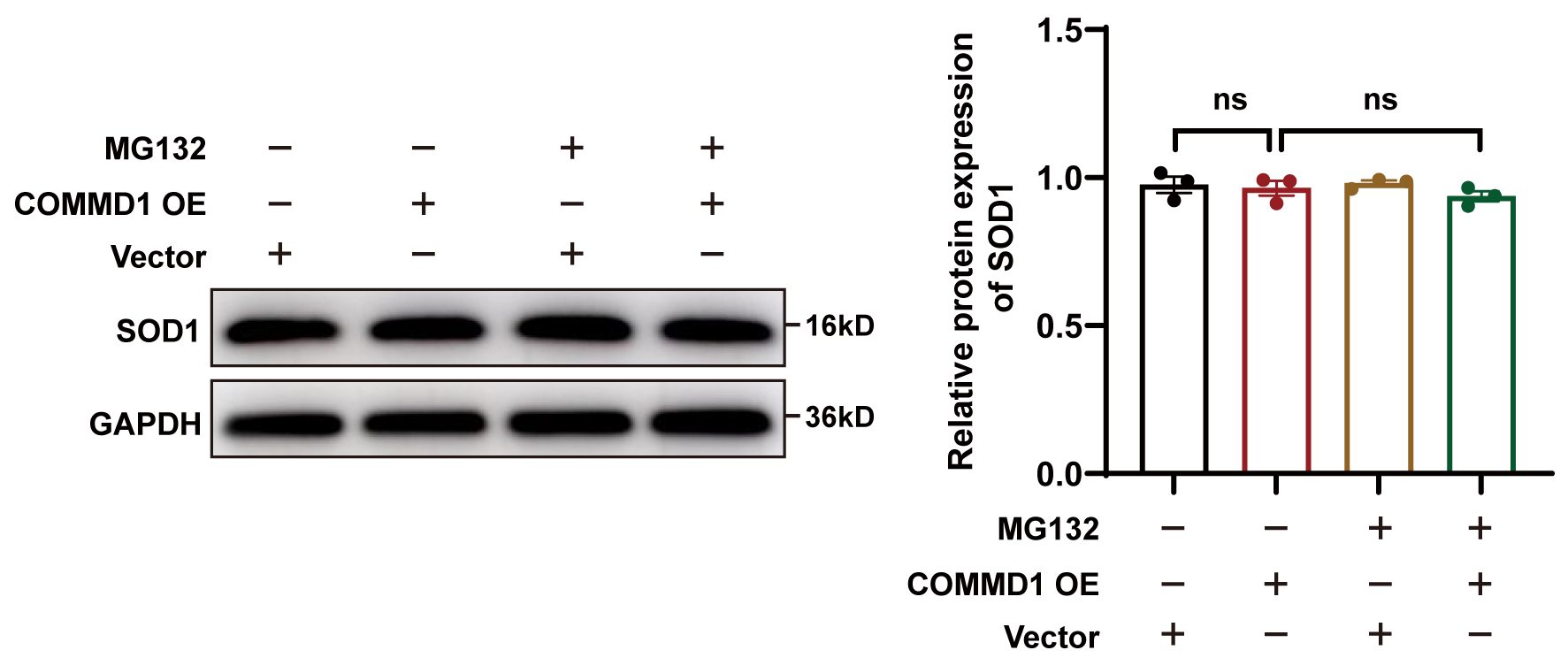

Supplement: Supplementary file 10 — Supporting File 10: advs76391‐sup‐0010‐FigureS9.tif. [file ADVS-9999-e76391-s007.tif]

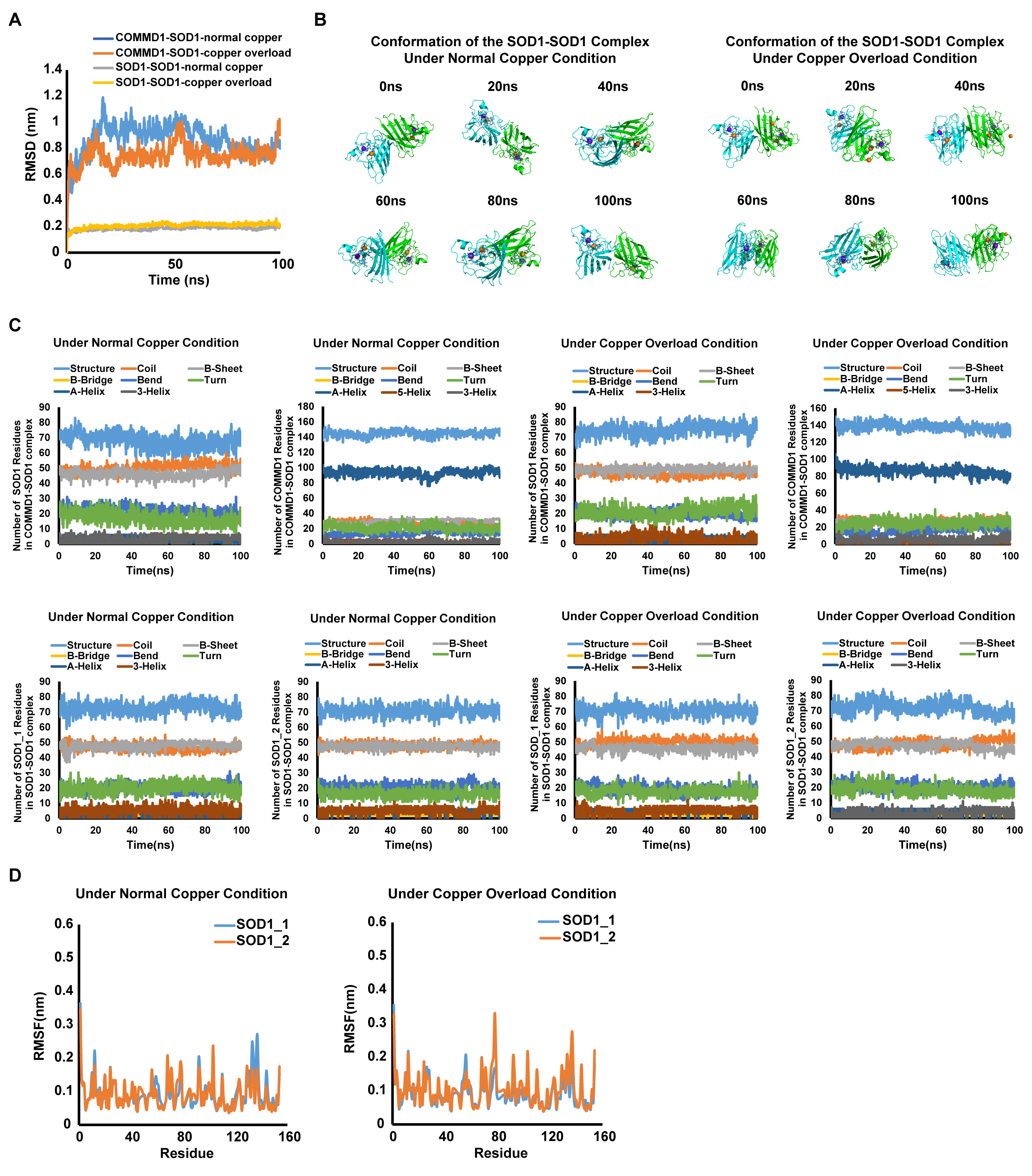

Supplement: Supplementary file 11 — Supporting File 11: advs76391‐sup‐0011‐FigureS10.tif. [file ADVS-9999-e76391-s011.tif]

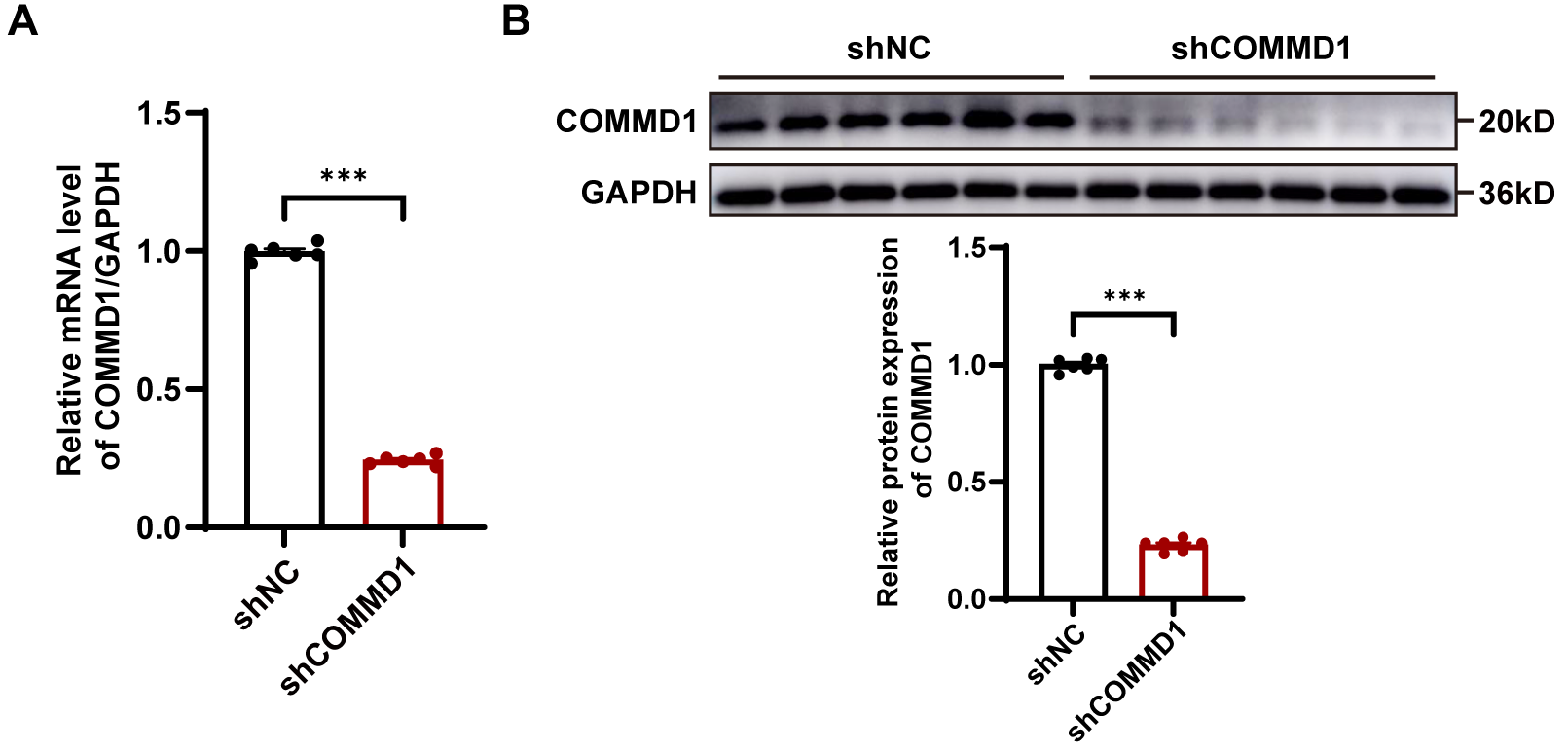

Supplement: Supplementary file 12 — Supporting File 12: advs76391‐sup‐0012‐FigureS11.tif. [file ADVS-9999-e76391-s002.tif]
